# Prediction Models for Maternal and Offspring Short‐ and Long‐Term Outcomes Following Gestational Diabetes: A Systematic Review

**DOI:** 10.1111/obr.13934

**Published:** 2025-05-04

**Authors:** Yasmina Al Ghadban, Nerys M. Astbury, Abdallah Kurdi, Ankita Sharma, Beatrice Ope, Tzu‐Ying Liu, Lucy MacKillop, Huiqi Y. Lu, Jane E. Hirst

**Affiliations:** ^1^ Nuffield Department of Women's and Reproductive Health University of Oxford Oxford UK; ^2^ Nuffield Department of Primary Care Health Sciences University of Oxford Oxford UK; ^3^ NIHR Oxford Biomedical Research Centre, Oxford University Hospitals NHS Foundation Trust Oxford UK; ^4^ Department of Biochemistry and Molecular Genetics, Faculty of Medicine American University of Beirut Beirut Lebanon; ^5^ Faculty of Medicine, School of Public Health Imperial College London London UK; ^6^ The George Institute for Global Health Imperial College London London UK; ^7^ Nuffield Department of Population Health University of Oxford Oxford UK; ^8^ Oxford University Hospitals NHS Foundation Trust Oxford UK; ^9^ Institute of Biomedical Engineering, Department of Engineering Science University of Oxford Oxford UK

**Keywords:** gestational diabetes, prediction models, prognosis modeling

## Abstract

**Objectives:**

Gestational diabetes mellitus (GDM), affecting one in seven pregnant women worldwide, can have short‐ and long‐term adverse outcomes for both the mother and her baby. Despite a raft of prognostic models aiming to predict adverse GDM outcomes, very few have impacted clinical practice. This systematic review summarizes and critically evaluates prediction models for GDM outcomes, to identify promising models for further evaluation.

**Methods:**

We searched EMBASE, MEDLINE, Web of Science, CINAHL, and CENTRAL for studies that reported the development or validation of predictive models for GDM outcomes in mother or offspring (PROSPERO: CRD42023396697).

**Results:**

Sixty‐four articles detailing 103 developed and 12 validated models were included in this review. Of these, 45% predicted long term, 31% birth, and 23% pregnancy outcomes. Most models (87%) had a high risk of bias, lacking sufficient outcome events, internal validation, or proper calibration. Only eight models were found at low risk of bias.

**Conclusions:**

Our findings highlight a gap in rigorously developed prediction models for adverse GDM outcomes. There is a need to further validate existing models and evaluate their clinical utility to generate risk prediction tools capable of improving clinical decision‐making for women with GDM and their children.

## 1 Introduction

Gestational diabetes mellitus (GDM), glucose intolerance with first onset or recognition during pregnancy, is one of the most common pregnancy complications affecting up to one in seven pregnant women worldwide [1]. GDM can have short‐term adverse consequences for the mother and the baby, such as increased risk of preterm delivery, macrosomia, and admission to neonatal intensive care unit (NICU) [2]. Additionally, although most women diagnosed with GDM return to normoglycemia shortly after giving birth, the condition can have long‐term consequences for both the mother and the child. Women with GDM face an increased risk of developing Type 2 diabetes (T2DM): A meta‐analysis of 20 studies found that women with GDM were 7.4 times as likely to develop T2DM than women with normoglycemic pregnancies [3]. Women with GDM are also at a significantly higher risk of hypertension, obesity, and cardiovascular morbidity [4, 5]. In addition, their children are at a heightened risk of obesity, cardiovascular morbidity, and glucose intolerance later in life [6, 7, 8].

There are interventions that hold the potential to mitigate many GDM‐related complications, such as diet advice, lifestyle modification, and medications [9]. However, proper detection, treatment, and follow‐up programs are resource‐intensive and often face challenges with poor compliance. With increasing obesity and maternal age, GDM prevalence is likely to increase and continue to pose substantial burden on healthcare systems, necessitating the development of effective strategies for risk stratification, resource allocation, and personalized management [10].

**FIGURE 1:**
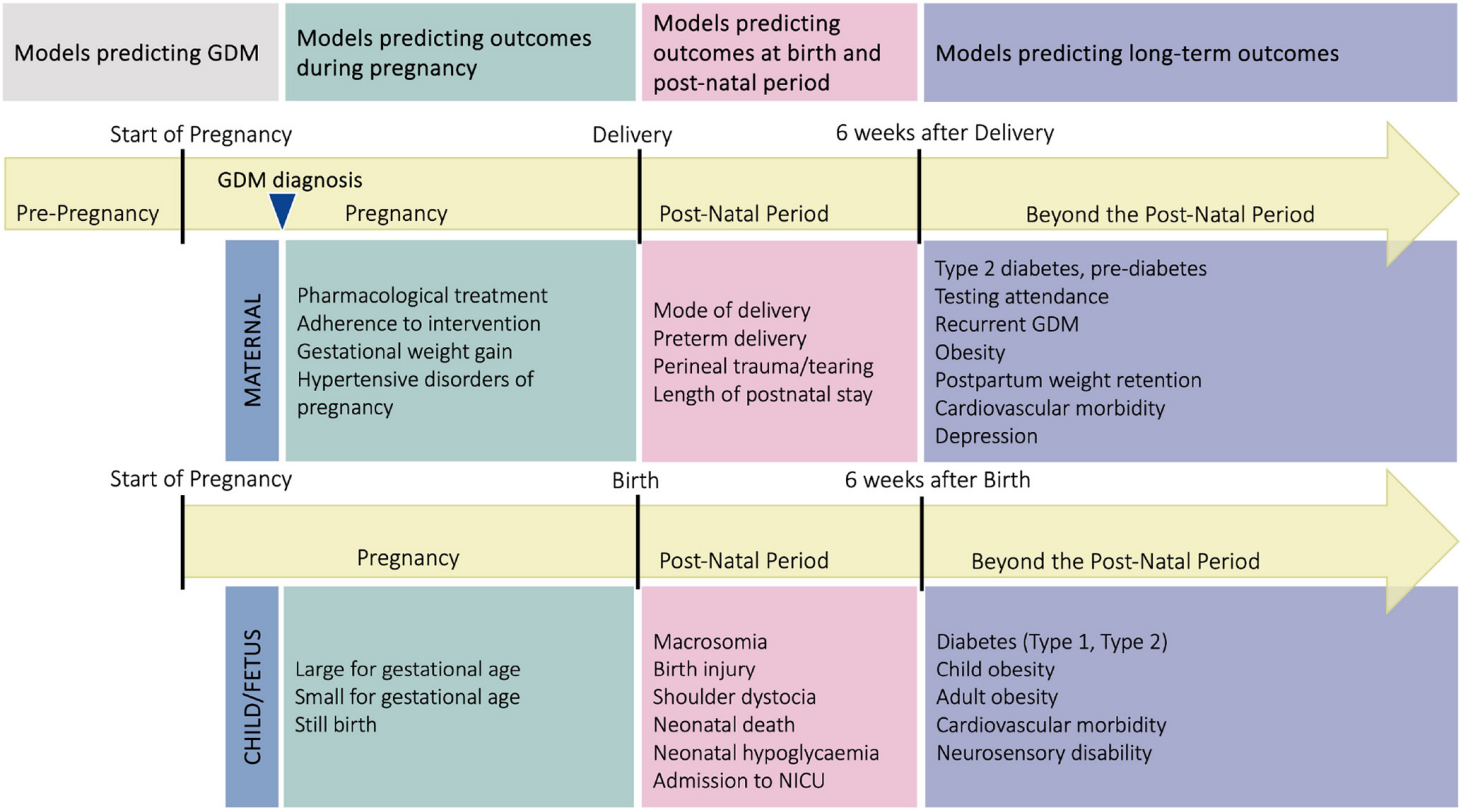
The use of prediction models at different stages of pregnancy: preconception, during pregnancy, at birth and in the postnatal period, and long‐term.

In recent years, researchers have increasingly turned to data‐driven prediction models combining various factors to estimate an individual's likelihood of developing a specific outcome. Prognostic models can be used to predict the risk of GDM preconception or in the early stages of pregnancy. Two recent reviews have studied and compared prognostic models predicting the risk of developing GDM [11, 12] and found that although several published models achieved acceptable discrimination and calibration, they varied in quality and lacked independent validation. After a diagnosis of GDM, prognostic models can also be used to predict the risk of GDM outcomes during pregnancy; at birth and in the postnatal period; and long term (Figure 1). Despite the growing interest and potential of prediction models for women with GDM, none to our knowledge is currently used widely in clinical practice. A systematic review of five prediction models for adverse outcomes during pregnancy in women with GDM pregnancy found that all models were at high risk of bias in their development, with a lack of external validation limiting their clinical utility [13]. However, given the fast development in this field, there has been further work because this review was conducted. Additionally, one of the most important consequences of GDM is the association with longer term cardiometabolic complications. Although several prediction models have been published in this area, to our knowledge, no review has critically appraised the quality and clinical utility of this evidence.

As such, the aim of this systematic review was to critically evaluate published prognostic models for the prediction of short‐ and long‐term outcomes associated with GDM, considering outcomes for both the woman and offspring. This synthesis will inform whether further testing of existing models is justifiable or the development of new models is needed before predictive models will make a measurable impact on clinical outcomes.

## 2 Methods

The systematic review was registered on PROSPERO, the International Prospective Register of Systematic Reviews (CRD42023396697), and follows the Preferred Reporting Items for Systematic reviews and Meta‐Analyses (PRISMA) statement [14].

### 2.1 Search Strategy

We searched EMBASE, MEDLINE, CENTRAL, Web of Science (core collection), and CINAHL. The search filters included keywords related to pregnancy, diabetes, and prognostic models and were developed based on previously validated search filters [15, 16]. No restrictions based on language were included in the strategy. Forward and backward citation searching was also performed for the studies included to improve the yield of the search strategies. An example search strategy is presented in Table S1. All databases were last searched on September 8, 2023.

### 2.2 Selection Criteria

The eligibility criteria were formulated with the PICOTS (Population, Intervention, Comparator, Outcomes, Timing, Setting) framework provided by the CHecklist for critical Appraisal and data extraction for systematic Reviews of prediction Modelling Studies (CHARMS) (Table 1) [17].

**TABLE 1:** Framing the review eligibility criteria with PICOTS.

PICOTS	
Type of model	1. Prognosis model studies without external validation in independent dataset 2. Prognosis model studies with external validation in independent dataset 3. External model validation studies with or without model updating
Population	Women with GDM as defined in the included studies and children born to mothers with GDM
Intervention	Prognostic model developed to predict maternal and child GDM outcomes
Comparator	Not applicable
Outcome	Maternal and child outcomes, listed in Figure 1, were selected based on outcomes studied in the literature [18] and a core outcome set developed for GDM [19]. Due to variations in outcome definitions, outcomes were collected as reported and defined in the included studies.
Timing	Models predicting pregnancy or birth outcomes will evaluate endpoints during the period between diagnosis with GDM until the end of the postnatal period (6 weeks after delivery). Models predicting long‐term outcomes will evaluate endpoints at defined months or years after delivery.
Setting	The intended use of prognostic models is to perform risk stratification in the assessment of pregnant women to inform healthcare professionals' decision making and resource allocation. As such, the models are designed for use in clinical settings.

Model development studies with or without validation and external model validation studies with or without model updating were included if they were predicting at least one outcome of interest, as a single endpoint or composite, as defined in the specific studies (Figure 1).

We excluded studies reporting models with a single predictor, studies investigating overall prognosis or prognostic factors without model development or validation, studies that included only pregestational diabetes or only twin pregnancies; and nonoriginal and non–peer‐reviewed studies. Studies with both GDM and pregestational diabetes women were included only if the models/outcomes are reported separately for women with GDM. There were no restrictions on the type of data used, study design, timing, or setting of data collection.

### 2.3 Selection Process

The results of the literature search were imported into Covidence, a systematic review management software, for deduplication and screening [20]. Title/abstract screening and full text screening were performed by two independent reviewers (YAG and AS), with a third reviewer (JEH) referred to for conflicts.

### 2.4 Data extraction and Critical Appraisal

Data extraction was informed by CHARMS [17], and critical appraisal and assessment of applicability were done using the Prediction model Risk Of Bias ASsessment Tool (PROBAST) [21]. A template for data extraction and risk of bias assessment in systematic reviews of predictive models informed by CHARMS and PROBAST was used [22].

Specifically, for model development, we extracted the following items: study design; outcome definition and measurement; candidate predictors definition, measurement, and method of selection; included predictors; sample size; number of events; handling and reporting of missing data; type of model; and methods and metrics used for model evaluation. For studies reporting validation, we extracted similar items but also extracted differences between the model development and validation regarding definition and measurement of predictors and outcomes, handling of continuous predictors and methods used to handle missing data.

Data extraction and critical appraisal were performed once for each development and validation of each distinct prediction model in a publication. In a deviation to the published protocol, data extraction and critical appraisal were completed by the lead reviewer (YAG) and verified by a second reviewer (AK, BO, and TYL) for all models. Uncertainties were discussed between reviewers and referred to a third (JEH) when an agreement could not be reached.

### 2.5 Data Synthesis

A meta‐analysis was not applicable given the heterogeneity of studies included and the lack of external validation studies available for any one model. Instead, data were synthesized qualitatively as reported in the protocol, using generated tables from the template of CHARMS and PROBAST [22].

## 3 Results

### 3.1 Study Selection

**FIGURE 2:**
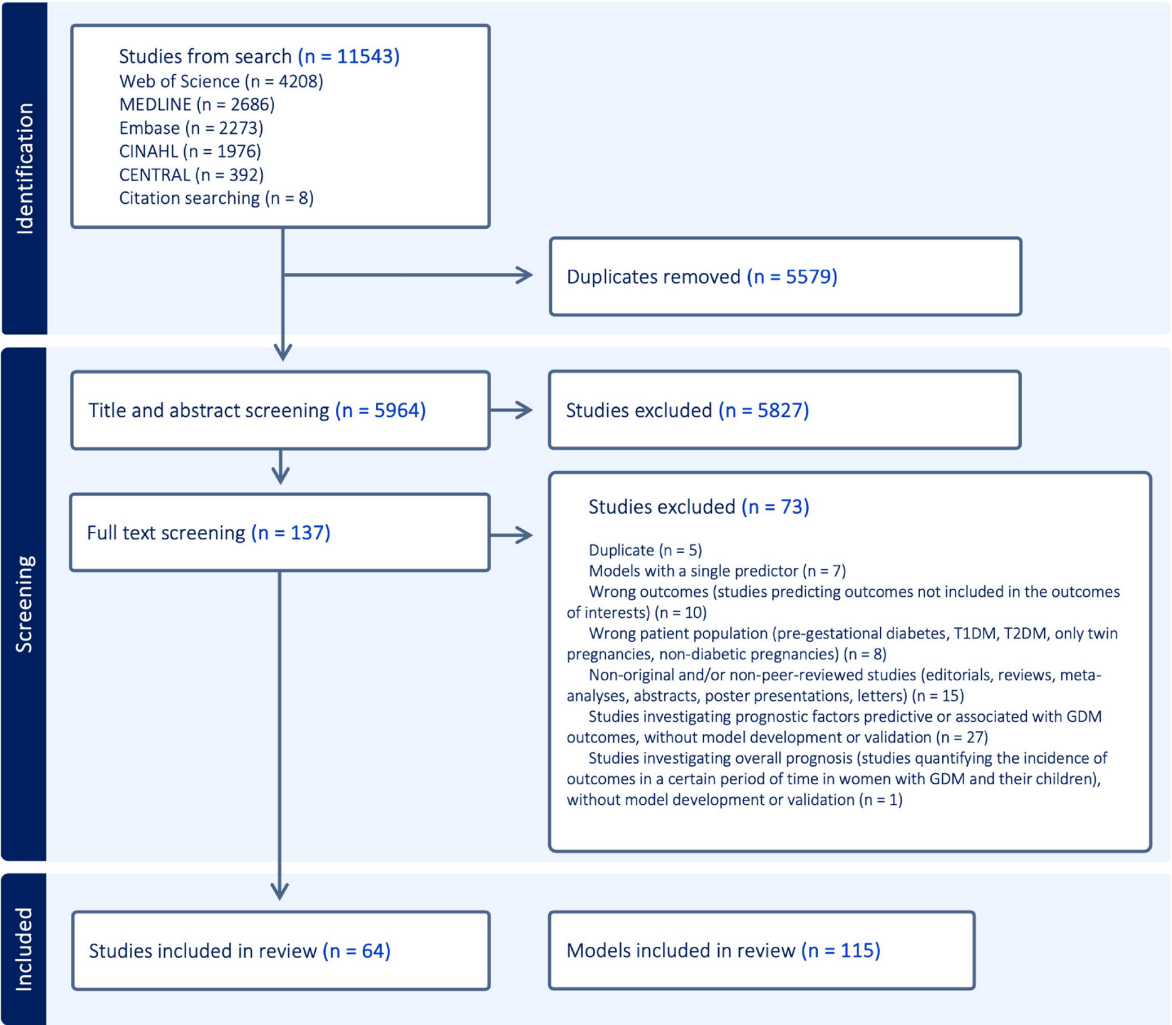
PRISMA flow diagram of study selection.

Our searches identified 11,543 records. After deduplication, 5964 studies were screened by title and abstract, and 137 studies were screened by full text screening. Sixty‐four studies, describing 115 models (103 model development and 12 external validation), were included in this review (Figure 2).

### 3.2 Included Study Characteristics

Of the 64 included studies, most were retrospective cohort studies (39/64; 61%), 16 (25%) were prospective cohort studies, 4 (6%) were nested case–control studies, and 2 (3%) were randomized trials. Forty‐one (64%) studies were single center studies.

Studies were from 24 different countries, with most represented countries (17/24, 71%) being high‐income countries according to the World Bank 2023 classification [23]. Nearly half of the studies (30/64; 47%) were based in Europe and North America and the Caribbean. A significant proportion of studies (25/64; 39%) originated from the Western Pacific region, whereas fewer than 10% of studies represented South America, Africa, the MENA region (Middle East and North Africa), or Southeast Asia. Diagnostic criteria for GDM varied across studies and regions, with the most common being the Carpenter & Coustan criteria [24] and the International Association of Diabetes in Pregnancy Study Group (IADPSG) criteria [25].

The overwhelming majority of studies (56/64; 88%) were model development studies without external validation. Seven studies (11%) reported both model development and external validation, and only one (2%) study described an external validation study without model development. The validation only article included several authors who were also on the paper that developed the model being evaluated. Thirty‐nine (61%) studies reported one model, whereas the remaining 25 (39%) studies reported multiple models. Studies reported more than one model for a range of reasons: external validation, use of different predictors or populations, prediction of different outcomes, or time of outcome ascertainment.

Long‐term outcomes were most common, representing just under half of all studies (29/64; 45%). Among maternal cardiometabolic complications, outcomes predicted included Type 2 diabetes in 19 studies (30%), glucose metabolism complications (postpartum glucose intolerance, prediabetes, and abnormal glucose tolerance) in 8 studies (13%), and lipid metabolism issues in 1 study (2%). Long‐term outcome studies exclusively focused on maternal health with no model predicting long‐term offspring outcome. Birth outcomes were predicted in 20 studies (31%) with macrosomia predicted in 10 studies. Eleven studies specifically targeted offspring birth outcomes and an additional five studies examining both maternal and offspring outcomes. Finally, pregnancy outcomes were predicted in 15 studies (23%) with 14 studies predicting treatment modality. Almost all studies reported single outcomes, with only three studies predicting composite outcomes.

Reporting of participant characteristics varied by study. Around half of the studies reported the number of nulliparous women (35/64; 55%) and the population's ethnic composition (32/64; 50%). Additionally, excluding studies that aimed to predict GDM treatment modality, 29/47 studies (62%) reported GDM treatment. One study included only women on glyburide [26], three studies did not include women receiving any pharmacological treatment [27, 28, 29], and one study women received no treatment for GDM (including medical nutrition therapy) as both clinicians and patients were blinded to the oral glucose tolerance test (OGTT) results [30].

The full details of included studies are available in Table 2.

**TABLE 2:** Characteristics of the studies included in the systematic review (*n* = 64).

Author, year	Source of data (sample size)	Enrolment period	Study setting	GDM diagnosis criteria	Participant characteristic^a^			Number of models (reasons for multiple models)	Outcome (s)
					Ethnicity	Nulliparous	Pharmacological treatment		
**Pregnancy outcomes**									
Phaloprakarn, 2009 [31]	Retrospective cohort (*n* = 813)	Jan 2003 to Feb 2008	Single center; Bangkok, Thailand	Universal screening; Carpenter & Coustan	92% Thai; 8% Southeast Asians	40.0%	34.1%	1	Preeclampsia
Badr, 2022 [32]	Retrospective cohort (*n* = 1298)	Jan 2018 to Dec 2018	Single center; Brussels, Belgium	IADPSG	85.0% Europe, North Africa, Middle East; 12.0% Sub‐Saharan Africa; 2.9% South or East Asia	27.3%	NR	4 (2 different screening used and external validation)	Treatment modality
Barnes, 2016 [33]	Retrospective cohort (*n* = 4015)	1992 to 2015	Single center; New South Wales, Australia	Universal screening; ADIPS 1998	33.7% East and South‐East Asian; 27.0% Middle Eastern; 22.5% European; 11.5% South Asian; 5.3% African or Pacific Islander	33.0%	32.4%	2 (external validation)	Treatment modality
Du, 2021 [34]	Retrospective cohort (*n* = 626)	May 2016 to Dec 2019	Single center; Sheng Jing Hospital, China	IADPSG	100% Chinese	81.5%	30.0%	1	Treatment modality
Eleftheriades, 2021 [35]	Prospective cohort (*n* = 775)	2010 to 2018	Single center; Athens, Greece	Universal screening; IADPSG	NR	NR	16.8%	2 (external validation)	Treatment modality
Ford, 2022 [36]	Retrospective cohort (*n* = 2048)	Jan 2016 to Dec 2017	Single center; Melbourne, Australia	Universal screening; IADPSG	NR	29.5%	31.6%	1	Treatment modality
Harper, 2016 [26]	Retrospective cohort (*n* = 220)	Jan 2007 to Dec 2013	Single center; Alabama, USA	Universal screening; Carpenter & Coustan	51.4% Black; 12.3% White; 28.6% Hispanic; 41.8% Other	27.7%	100.0%	1	Treatment modality
Koefoed, 2023 [37]	Retrospective cohort (*n* = 1104)	2012 to 2017	Single center; Denmark	Selective screening; Danish Guidelines	86.9% Caucasian; 12.1% Afro‐Caribbean; 1.1% Other	44.4%	25.5%	1	Treatment modality
Liao, 2022 [38]	Retrospective cohort (*n* = 27,240)	Jan 2007 to Dec 2016	Multicenter; California, USA	Universal screening; Carpenter & Coustan	22.7% White; 28.1% Hispanic; 4.3% African American; 40.3% Asian/Pacific Islander; 4.6% Other	40.8%	37.8%	8 (different predictors and external validation)	Treatment modality
Mendez‐Figueroa, 2014 [39]	Retrospective cohort (*n* = 367)	Jun 2006 to Dec 2011	Single center; Rhode Island, USA	Carpenter & Coustan	23.5% White; 13.7% African American; 15.0% Asian; 39.0% Hispanic; 8.7% Other	18.3%	38.9%	1	Treatment modality
Much, 2015 [40]	Retrospective cohort (*n* = 856)	1998 to 2010	Single center; Munich, Germany	IADPSG	NR	NR	21.4%	1	Treatment modality
Souza, 2018 [41]	Retrospective cohort (*n* = 408)	2012 to 2015	Single center; Sao Paulo, Brazil	IADPSG	NR	NR	33.1%	1	Treatment modality
Velardo, 2021 [42]	Retrospective cohort (1543)	2014 to 2019	Multicenter; NHS Trusts, UK	Varies by center	NR	NR	NR	1	Treatment modality
Yerlikaya, 2018 [43]	Retrospective cohort (*n* = 203)	May 2015 to Jan 2017	Single center; Vienna, Austria	Universal screening; IADPSG	NR	27.6%	46.8%	3 (different predictors)	Treatment modality
Zaccara, 2023 [44]	Retrospective cohort (*n* = 869)	Jan 2012 to Mar 2020	Single center; Sao Paulo, Brazil	Universal screening; IADPSG	NR	NR	16.7%	1	Treatment modality
**Birth outcomes**									
Pintaudi, 2018 [45]	Retrospective cohort (*n* = 2736)	Jan 2012 to May 2015	Multicenter; Italy	Selective screening; IADPSG	Caucasian 44.8%	45.3%	41.3%	1	Adverse outcomes composite
Cooray, 2022 [46]	Retrospective cohort (*n* = 1747)	Jul 2017 to Jun 2018	Multicenter; Melbourne, Australia	Universal screening; IADPSG	19.6% Caucasian; 35.3% Southern and Central Asian; 13.8% East Asian; 5.2% African; 3.3% Oceanian not Australian; Other 22.9%	38.2%	36.6%	2 (external validation)	Adverse outcomes composite
Park, 2015 [47]	Retrospective cohort (*n* = 802)	Mar 2001 to Apr 2013	Single center; Seoul, South Korea	Carpenter & Coustan	NR	NR	NR	1	Adverse outcomes composite
Lu, 2022 [48]	Randomized trial (*n* = 155)	Sept 2013 to Jun 2015	Single center; Oxford, UK	IADPSG	NR	NR	NR	2 (different predictors)	Cesarean delivery
Phaloprakarn, 2020 [31]	Retrospective cohort (*n* = 385)	Jan 2011 to Dec 2014	Single center; Bangkok, Thailand	Universal screening; Carpenter & Coustan	NR	41.6%	12.2%	2 (external validation)	Cesarean delivery
Ramos, 2023 [49]	Retrospective cohort (*n* = 3570)	Jan 2022 to Mar 2013	Single center; USA	Carpenter & Coustan	62.4% White; 7.2% Black; 5.9% Asian; 24.5% Other	55.2%	24.9%	1	Cesarean delivery
Kim, 2022 [50]	Retrospective cohort (*n* = 660)	2006 to 2013	Multicenter; South Korea	Carpenter & Coustan	NR	NR	NR	1	Large for gestational age
Kang, 2019 [51]	Retrospective cohort (*n* = 1891)	Jan 2010 to Dec 2017	Single center; Nantong, China	NR	NR	NR	NR	2 (different predictors)	Macrosomia
Odinokova, 2019 [52]	Prospective cohort (*n* = 220)	2017 to 2018	Moscow, Russia	IADPSG	100% Caucasian	NR	NR	1	Macrosomia
Sun, 2020 [53]	Prospective cohort (*n* = 64)	Jun 2017 to Apr 2018	Single center; Hangzhou, China	IADPSG	100% Chinese Han	76.4%	NR	1	Macrosomia
Sun, 2023 [54]	Retrospective cohort (*n* = 991)	Nov 2020 to Feb 2022	Single center; Shanxi, China	IADPSG	NR	NR	9.3%	1	Macrosomia
Tomlinson, 2018 [55]	Retrospective cohort (*n* = 275)	Mar 2010 to May 2012	Massachusetts, USA	Carpenter & Coustan	82.0% White; 5.0% Black; 12.0% Other	51.0%	NR	1	Macrosomia
Tomlinson, 2021 [56]	Retrospective cohort (*n* = 477)	Jan 2015 to Jun 2018	Perinatal database, St Louis, USA	Carpenter & Coustan	54.0% White; 40.0% Black; 6.0% Other	30.0%	NR	1	Macrosomia
Yuan, 2022 [57]	Unclear (*n* = 40)	Jul 2019 to Jun 2021	Multicenter; Jiangsu, China	IADPSG	NR	NR	NR	1	Macrosomia
Yuan, 2023 [27]	Retrospective cohort (*n* = 88)	Jan 2020 to Jun 2020	Single center; Nanjing, China	IADPSG	100% Han Chinese	70.5%	0%	1	Macrosomia
Zhang, 2023 [28]	Retrospective cohort (*n* = 322)	Oct 2020 to Oct 2021	Single center; Shantou City, China	Universal screening; IADPSG	NR	NR	0%	1	Macrosomia
Zou, 2021 [58]	Retrospective cohort (*n* = 783)	Oct 2019 to Oct 2020	Single center; Qingdao, China	IADPSG	NR	NR	NR	1	Macrosomia
Huang, 2023 [59]	Retrospective cohort (*n* = 564)	Jan 2017 to Jan 2020	Single center; Wenzhou, China	IADPSG	NR	63.5%	10.5%	2 (external validation)	Preterm delivery
Mukherjee, 2023 [60]	Retrospective cohort (*n* = 627)	2015 to 2016	UK	Selective screening; IADPSG	NR	NR	33.2%	7 (different outcomes)	Preterm birth, hypoglycemia, hyperbilirubinemia, NICU, macrosomia, shoulder dystocia, composite
McIntyre, 2018 [30]	Prospective cohort (*n* = 1248)	Jul 2000 to Apr 2006	Single center; South Brisbane, Australia	ADIPS 1991; IADPSG; Carpenter & Coustan; NICE 2015	100% Caucasian	54.5%	NR	6 (different outcomes)	Cesarean delivery, birth injury, LGA, adiposity, hyperinsulinemia, hypoglycemia
**Long‐term outcomes**									
Jotic, 2023 [29]	Prospective cohort (*n* = 147)	Jan 2017 to Dec 2021	Single center; Serbia	IADPSG	NR	NR	0%	1	Dyslypidemia
Hahn, 2023 [61]	Retrospective cohort (*n* = 159)	Jan 2014 to Sept 2020	Single center; Germany	IADPSG	NR	NR	59.7%	1	GDM recurrence
Schwartz, 2016 [62]	Retrospective cohort (*n* = 788)	1991 to 2012	Single center; Israel	Universal screening; Carpenter & Coustan or NDDG	52.8% Jews; 47.2% Arabs	48.5%	34.1%	1	GDM recurrence
Bengtson, 2022 [63]	Prospective cohort (*n* = 203)	Jan 2017 to Jul 2018	Single center; Rhode Island, USA	Carpenter & Coustan	48.3% White; 10.3% Non‐Hispanic black; 30.5% Hispanic; 10.8% Other	NR	57.1%	1	Postpartum glucose intolerance
Kondo, 2018 [64]	Retrospective cohort (*n* = 123)	Apr 2012 to Apr 2017	Single center; Yamaguchi‐City, Japan	Universal screening; IADPSG	NR	NR	22.0%	3 (different predictors)	Postpartum glucose intolerance
Bartakova, 2021 [65]	Retrospective cohort (*n* = 244)	2011 to 2013	Single center; Czech Republic	Unclear	100% Caucasian	44.3%	37.3%	1	Postpartum glucose intolerance
Pei, 2023 [66]	Prospective cohort (*n* = 133)	Jan 2018 to Dec 2020	NR	IADPSG	NR	NR	NR	1	Impaired glucose tolerance remission
Muche, 2020 [67]	Prospective cohort (*n* = 112)	Mar 2018 to Mar 2019	Multicenter; Gonda, Ethiopia	Universal screening; IADPSG	NR	NR	6.2%	1	Postpartum prediabetes
Periyathambi, 2022 [68]	Retrospective cohort (*n* = 607)	Jan 2016 to Dec 2019	Single center; Nuneaton, UK	Selective screening; NICE 2015	79.2% White European; South Asian 11.1%; Other 9.7%	43.9%	NR	1	Return for testing
Ingram, 2017 [69]	Retrospective cohort (*n* = 165)	Jan 2007 to Jun 2009	Single center; Tasmania	Universal screening; ADIPS 1998	86.6% Caucasian; 4.8% Aboriginal and Torres Strait; 8.5% Other	NR	60.6%	3 (different outcome)	Return for testing, abnormal glucose tolerance
Barden, 2013 [70]	Prospective cohort (*n* = 150)	1998 to 2001	Multicenter; Perth, Western Australia	ADIPS 1998	74.7% Caucasian; 25.3% Asian/aboriginal/middle‐eastern	18.7%	NR	1	Type 2 diabetes
Lin, 2011 [71]	Retrospective cohort (*n* = 607)	NR	Single center; Taipei, Taiwan	Universal screening; NDDG	NR	NR	NR	1	Type 2 diabetes
Man, 2021 [72]	Randomized trial (*n* = 317)	1996 to 2001	Multicenter; USA	NR	59.3% Caucasian; 19.9% African American; 17.0% Hispanic; 3.8% Other	NR	33.1%	1	Type 2 diabetes
Ignell, 2016 [73]	Prospective cohort (*n* = 362)	2003 to 2005	Multicenter; Skane, Southern Sweden	Universal screening; Modification to WHO 1999	16.0% non‐European	NR	9.1%	2 (different population)	Type 2 diabetes
Allalou, 2016 [74]	Nested case–control (*n* = 244)	Sep 2008 to Dec 2011	Multicenter; California, USA	Carpenter & Coustan	17.2% White; 30.7% Asian; 11.1% Black; 39.3% Hispanic; 1.6% Other	35.2%	46.3%	3 (different predictors)	Type 2 diabetes
Joglekar, 2021 [75]	Prospective cohort (*n* = 82)	Jun 2003 to Dec 2005	Single center; Australia	ADIPS 1998	NR	NR	48.1%	2 (different predictors)	Type 2 diabetes
Khan, 2019 [76]	Nested case–control (*n* = 140)	Sept 2008 to Dec 2011	Multicenter; California, USA	Carpenter & Coustan	64.3% Hispanic; 35.7% Asian	23.6%	42.2%	1	Type 2 diabetes
Kwak, 2013 [77]	Prospective cohort (*n* = 395)	Jan 1996 to Feb 2003	Single center; Seoul, South Korea	Universal screening; NDDG	NR	NR	28.4%	4 (different predictors)	Type 2 diabetes
Lai, 2020 [78]	Nested case–control (*n* = 337)	Sept 2008 to Dec 2011	Multicenter; California, USA	Carpenter & Coustan	17.9% White; 29.8% Asian; 11.7% Black; 39.4% Hispanic; 1.2% Other	35.2%	46.3%	1	Type 2 diabetes
Lappas, 2015 [79]	Prospective cohort (*n* = 104)	Jun 2003 to Dec 2005	Single center; Australia	ADIPS 1998	NR	NR	48.1%	3 (different predictors)	Type 2 diabetes
Lappas, 2016 [80]	Prospective cohort (*n* = 95)	Jun 2003 to Dec 2005	Single center; Australia	ADIPS 1998	NR	NR	51.0%	3 (different predictors)	Type 2 diabetes
Lappas, 2018 [81]	Prospective cohort (*n* = 98)	Jun 2003 to Dec 2005	Single center; Australia	ADIPS 1998	NR	NR	49.5%	3 (different predictors)	Type 2 diabetes
Zhang, 2021 [82]	Nested case–control (*n* = 216)	Sept 2008 to Dec 2011	Multicenter; California, USA	Carpenter & Coustan	16.2% White; 31.5% Asian; 8.3% Black; 43.1% Hispanic; 0.9% Other	NR	42.6%	1	Type 2 diabetes
Li, 2018 [83]	Retrospective cohort (*n* = 1263)	2005 to 2009	Single center; Tianjin, China	Universal screening; WHO 1999	100% Chinese	NR	NR	2 (different time for outcomes)	Type 2 diabetes
Elnour, 2006 [84]	Unclear (*n* = 165)	Dec 2001 to Jun 2002	Single center; UAE	Universal screening; Carpenter & Coustan	NR	NR	NR	1	Type 2 diabetes
Kohler, 2016 [85]	Prospective cohort (*n* = 304)	1989 to 1999	Multicenter; Germany	German Diabetes Association	NR	NR	33.5%	1	Type 2 diabetes
Marozas, 2018 [86]	NR (*n* = 151)	NR	NR	NR	NR	NR	14.4%	2 (different outcomes)	Type 2 diabetes and impaired metabolism
Cormier, 2014 [87]	Retrospective cohort (*n* = 214)	Oct 2009 to Aug 2012	Quebec City, Canada	NR	93.6% White	NR	NR	2 (different outcomes)	Type 2 diabetes and prediabetes
Ukah, 2022 [88]	Retrospective cohort (*n* = 90,143)	Apr 1989 to Mar 2016	Multicenter; Quebec, Canada	Varies by center	NR	60.3%	NR	2 (different time for outcomes)	Type 2 diabetes complications

^a^ When possible, in studies that reported patient characteristics for each subgroup, data were converted to get a value for the complete sample.

Abbreviations: ADIPS, Australasian Diabetes in Pregnancy Society; IADPSG, International Association of Diabetes in Pregnancy Study Groups; LGA, large for gestational age; NDDG, National Data Diabetes Group; NICE, National Institute for Health and Care Excellence; NICU, Neonatal Intensive Care Unit; NR, not reported; OGTT, Oral Glucose Tolerance Test; WHO, World Health Organization.

### 3.3 Sample Size

In 93 of 103 developed models (90%), data on the number of events and candidate predictors allowed EPV calculations, which ranged from 0.7 to 151.3 with a median of 5.5. Fewer than half (45/93; 48%) of these models met an EPV ≥ 10, and only a third (32/93; 34%) met an EPV ≥ 20. For the 12 validated models, four included fewer than 100 participants with the outcome.

### 3.4 Missing Data

An overwhelming majority did not report the number of missing data (93/115; 81%) or the handling of missing data (49/115; 43%). Of the models that reported how missing data were handled, over half (39/66; 59%) used complete‐case analysis, 18/66 (27%) used multiple imputation, and 5/66 (8%) used single imputation.

### 3.5 Model Development

The included studies described 103 developed models with varying model development methods (Table S2). The sample size used to develop the prediction models ranged from 40 to 90,143 with a median of 322, and the number of events ranged from 4 to 10,289 with a median of 78. Most models used logistic regression (74/103; 72%) and 15 (15/103, 15%) used machine‐learning methods. Among the 15 machine learning models, tree‐based models were the most common, with six models employing techniques such as decision trees, CART (Classification and Regression Trees), random forest, and RECPAM (REcursive Partition and AMalgamation). One study used a probabilistic model, specifically the Naive Bayes classifier. Two models were ensemble models, utilizing a voting meta‐algorithm to combine top‐performing classifiers. Neural networks were used in two models, applying deep learning techniques to capture complex relationships in GDM‐related data. Lastly, specialized methods were used in four studies, including cluster analysis, a filtered classifier, AIRS (Artificial Immune Recognition System), and OPLS‐DA (Orthogonal Partial Least Squares Discriminant Analysis) for classification tasks. From the 45 models predicting outcomes determined after months or years of follow up, only 7/45 (16%) models used Cox regression, a survival analysis method that can account for loss to follow up. In the 84 models that used regression‐based methods, only nine models (11%) applied shrinkage techniques to alleviate overfitting. Additionally, less than half (35/84; 42%) reported regression coefficients, and only 20/84 (24%) reported the full model equation including the intercept or baseline survival.

### 3.6 Predictors

In model development, 47/103 (46%) models selected candidate predictors based on univariable associations, whereas 28/103 (27%) selected candidate predictors based on existing knowledge.

**FIGURE 3:**
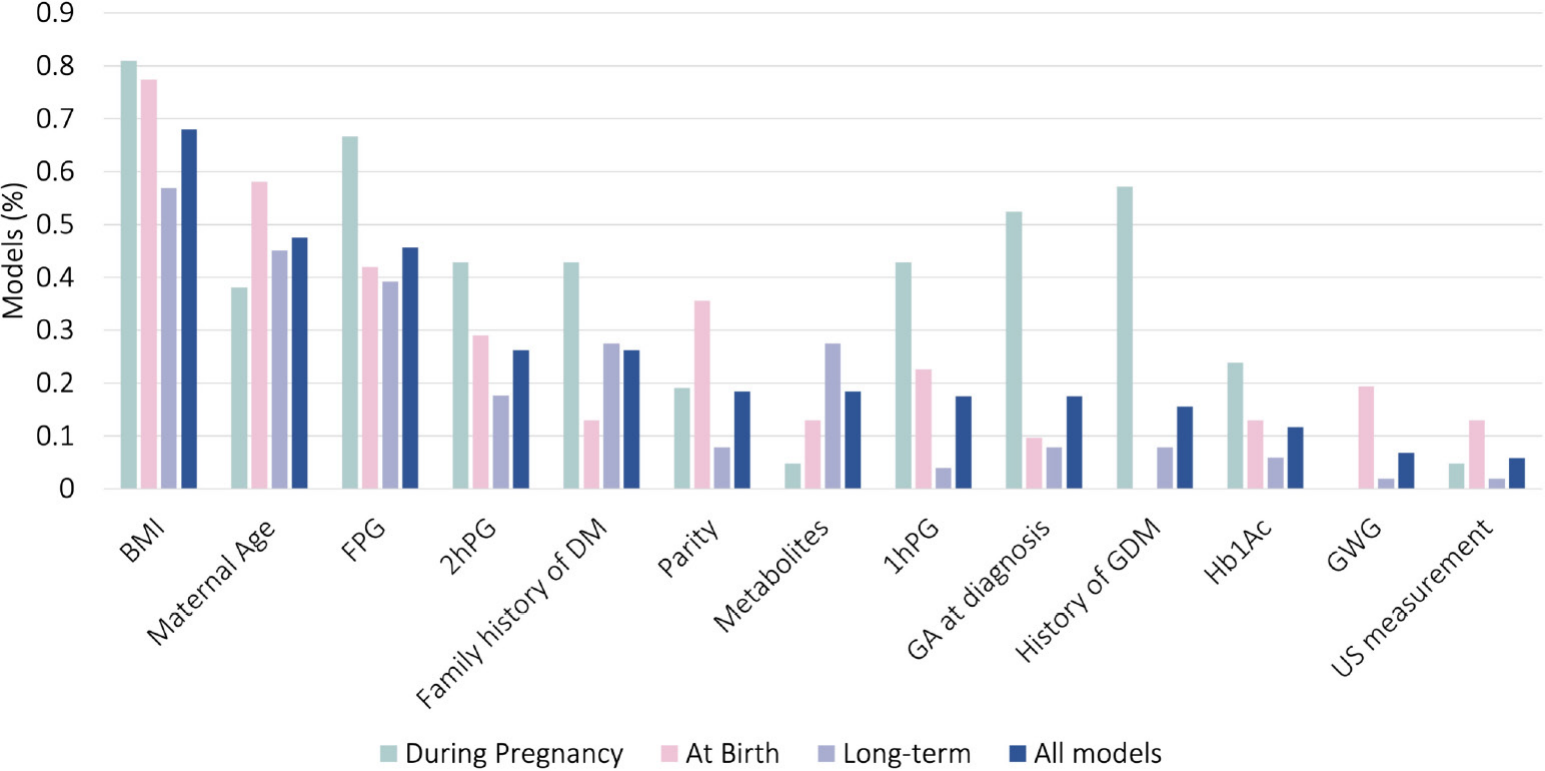
Bar graph showing the most common predictors included in the development of the models (*n* = 103) by type of model.

The number of predictors in the model ranged from 2 to 20, with a median of 5. A variety of candidate predictors were selected across models (Table S2). Clinical factors routinely available from standard electronic health records (BMI, age, ethnicity, parity, and medical history) and blood glucose measures recorded at or around the time of GDM diagnosis (Hb1Ac, OGTT results) were the most used. However, 29 (28.2%) models also used predictors not typically available in clinical practice, such as omics (proteomics, metabolomics, and transcriptomics), genetic factors, or digital daily blood glucose readings. The single most commonly used predictor was BMI, included in 70 models (68%), followed by maternal age included in 49 models (48%), and fasting plasma glucose included in 47 models (46%) (Figure 3).

Studies typically reported the mean, standard deviation, or interquartile range of continuous predictors. Only 10 models (10/103; 10%) reported the range of any continuous predictors. Additionally, only half of models (51/103; 50%) handled continuous variables without categorization. In 21/27 (78%) of models that reported categorizing continuous variables, cut points were chosen by selecting the thresholds that result in the highest predictive performance, rather than by predefining thresholds.

### 3.7 Performance Metrics

Calibration metrics were reported in 30 models (26%). Among these, nine models (30%) employed the Hosmer–Lemeshow goodness of fit test. Only one model reported the calibration slope, whereas 20 included a calibration plot. *R* ^2^ values were reported in three models. Discrimination, measured through the C‐statistic (area under the ROC curve), was reported in 108 models (94%). Additionally, 60% of models (69/115) presented the ROC curve, but only 16% (11/69) labeled cut‐off points to determine sensitivity and specificity, whereas 51% (59/115) reported these metrics. Clinical utility—assessing the actual usefulness in decision‐making—was examined for 10/115 (9%) models using decision curve analysis (DCA), a novel method to calculate the net‐benefit of a model in comparison to default strategies such as treating all or none.

### 3.8 Model Presentation

Thirty‐five models (35/103; 34%) presented an alternative model presentation. Seventeen models (16/103; 16%) were presented as a score system, a simplified format where predictor coefficients are converted to integers and summed together. Eleven models (11/103; 11%) presented their model using a nomogram, a graphical representation that displays the associations between predictors and response in a multivariable model and enable users to estimate the outcome probability for each patient. The remaining models were presented using an online calculator or regression trees. Only 5/35 (14%) models presented both the full model equation and the simplified model.

### 3.9 Model Validation

Most models lacked internal or external validation. Of the 103 developed models, 43 (42%) included one form of internal validation. Nine of these used random sample splitting between development and validation, whereas 34 applied cross‐validation or bootstrap methods. External validation was reported in 12 models (12%). Clinical utility was evaluated in 10 models (9%), with decision curve analysis (DCA) applied to assess net benefit.

Of the 12 externally validated models, seven had an alternative presentation, but it was unclear whether the full model or the alternative model was validated. Nine of these models (9/12; 75%) used temporal validation, evaluating their model in data from the same center but from a different time period. Only three models (25%) were validated in an independent sample, and none were validated by independent researchers. All studies reported the sample size used to validate the model (range: 168 to 3234; median: 716), but two studies did not report the number of events. For validation studies that reported the number of events, the median number of events was 368 (range 628–1528). For 10/12 models, the characteristics of both the development and validation cohorts were reported, allowing a comparison of case mix between the two.

### 3.10 Risk of Bias and Applicability

**FIGURE 4:**
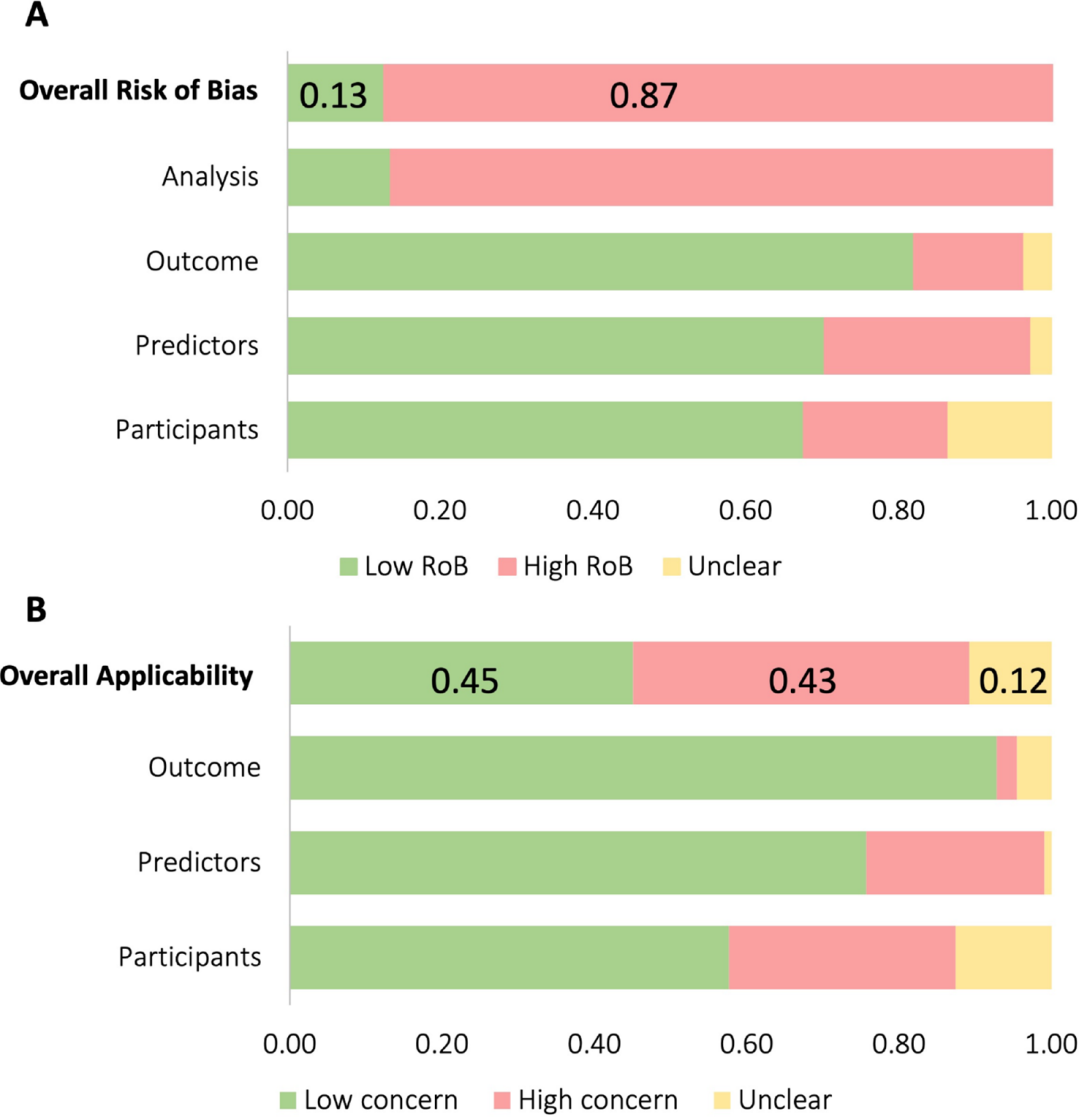
Summary of risk of bias (A) and applicability (B) assessment.

Most models (100/115; 87%) had an overall high risk of bias (Figure 4A). Low risk of bias was observed across the outcome, predictors, and participants domains; however, 99 models exhibited high risk of bias in the analysis domain. Less than half of models (52/115; 45%) were applicable to the research question (Figure 4B). Table S3 includes individual PROBAST domain ratings across all development and external validation analyses.

### 3.11 Gray Literature

We identified 13 conference abstracts relevant to this review [89, 90, 91, 92, 93, 94, 95, 96, 97, 98, 99, 100, 101]. The findings of six of these abstracts were subsequently published in peer‐reviewed articles included in this review [33, 46, 49, 50, 72, 75]. The limited information available in the abstracts hindered our ability to conduct adequate data extraction and critical appraisal. However, with the information available, we found similar methodological issues as in the included models such as inadequate evaluation of calibration, lack of internal validation, and small sample sizes.

## 4 Discussion

### 4.1 Summary of Findings

This systematic review is the largest review to date of prognostic models, which aim to determine the risk of adverse outcomes in women affected by GDM and her offspring. We included 64 studies, describing 115 models. The most common outcomes predicted were (i) the longer term risk of developing diabetes for the mother; (ii) birth outcomes, with a focus on macrosomia; and (iii) GDM treatment modality during pregnancy (diet or medication).

The regional distribution of studies revealed a concentration in Europe, North America, and the Western Pacific, where GDM prevalence is 7.2%, 7.8%, and 14.7% [1]. However, other regions with some of the highest reported GDM incidence rates—such as MENA (27.6%) and Southeast Asia (20.8%)—are underrepresented in the literature, with fewer than 10% of studies originating from these areas [1]. This imbalance may limit the generalizability of prediction models and underscores the need for more research in regions with a high GDM burden to ensure broader applicability and relevance across diverse populations.

Additionally, a significant gap was identified for models addressing long‐term health outcomes both for children born to mothers with GDM and for maternal cardiovascular risks. Children exposed to GDM are at higher risk for obesity, metabolic syndrome, and Type 2 diabetes [6, 7, 8], yet few models account for these intergenerational risks, reducing opportunities for early intervention that could improve long‐term health outcomes. Similarly, models that predict long‐term cardiovascular outcomes for women with GDM are limited, despite the known association between GDM and increased risks of hypertension and cardiovascular disease [4, 5]. Developing models that incorporate these risks could support preventive strategies for both mother and child, ultimately enhancing lifetime care through targeted interventions and comprehensive management of long‐term health risks associated with GDM.

Together, these gaps in regional representation, long‐term offspring outcome modeling, and models of cardiovascular outcomes highlight important limitations in the current body of GDM prediction research. Addressing these areas in future studies will be essential to develop more comprehensive, generalizable, and clinically useful models that support individualized care across diverse settings and patient populations.

### 4.2 Bias and Methodological Limitations

Overall, we found that most models are not suitable for clinical use in their current form due to major methodologic limitations. Of the 115 models, eight unique models were found to be at low risk of bias. These models predicted treatment modality [38], a composite of adverse outcomes [46], Type 2 diabetes complications [88], and primary cesarean section [49, 102]. External validation in different populations is needed to determine real‐world performance and impact.

These methodological challenges are consistent with findings in GDM research [13, 103] and other fields, such as of psychiatry, tuberculosis, and asthma [104, 105, 106], where prediction models frequently exhibit high risk of bias and inadequate calibration. The key methodological issues identified were as follows:

1. Sample size: Many models were developed with a low EPV, with fewer than half meeting the EPV ≥ 10 criterion and even fewer reaching the recommended EPV ≥ 20. These limitations raise concerns about overfitting, which can lead to overly optimistic model performance that fails to generalize to broader populations. To enhance the reliability of future models, studies should move beyond a one‐size‐fits‐all EPV threshold. Instead, sample size requirements should be calculated based on the specific complexity of each model and the expected predictive accuracy [107, 108]. Adopting these tailored sample size calculations will help produce more robust and clinically applicable models across diverse patient populations.
2. Handling of missing data: The reporting and handling of missing data were frequently insufficient, with over half of the models relying on complete‐case analysis. This approach can introduce significant bias by selectively excluding certain patients, resulting in a nonrandom subset of the study population, which can lead to skewed predictor‐outcome associations and biased model performance. Multiple imputation is regarded as the most appropriate method for handling missing data in prediction model development and validation [109, 110, 111]. It minimizes bias by estimating the missing values based on other observed data, thereby preserving the overall structure of the dataset and providing more accurate estimates of model performance, including correct standard errors and *p* values. Future studies should prioritize multiple imputation or other rigorous approaches for handling missing data, as these methods provide better precision and less bias. Additionally, it is crucial for authors to transparently report how missing data were handled and to provide comparisons between included and excluded participants to assess potential bias.
3. Predictor selection: In our review, many models relied on univariable analysis to select candidate predictors, an approach that selects predictors based solely on their individual statistical significance without considering how they interact with other variables. This method can result in omitting relevant predictors that may only show importance when adjusted for other factors or, conversely, including predictors based on accidental associations. Such selection practices increase the risk of both underfitting and overfitting, compromising the model's performance in new datasets. A more reliable approach to predictor selection emphasizes the use of nonstatistical methods grounded in established knowledge, clinical credibility, and practical considerations relevant to the model's intended setting. Predictors known to be significant in prior research should be retained, regardless of their statistical significance in a specific dataset, ensuring that the model incorporates clinically meaningful variables. Methods like backward selection or penalized regression can also help refine predictor sets in a multivariable context without relying on preliminary univariable testing, preserving predictor integrity while minimizing overfitting [112]. Additionally, using internal validation techniques to assess overfitting and optimize model performance is crucial when refining predictor sets [112, 113, 114]. Adopting these predictor selection strategies can improve model stability and enhance generalizability, ultimately producing models that are both simpler and more clinically relevant without compromising predictive power.
4. Model evaluation: Our review identified calibration as a frequently neglected aspect in model evaluation, with many studies relying solely on the Hosmer–Lemeshow test. This test is limited in statistical power, especially with large datasets, and can be overly sensitive to sample size, often leading to misleading assessments of calibration quality. Calibration plots and calibration slopes are more informative alternatives, as they visualize and quantify the alignment between predicted and observed outcomes across the risk spectrum, offering a more nuanced understanding of model performance [115, 116]. In addition, cut‐off points on ROC curves were often overlooked. Without these measures, the interpretation of discrimination—the model's ability to differentiate between individuals with and without the outcome—remains incomplete. Reporting only the area under the ROC curve (AUC) can obscure important calibration issues, as a model with high discrimination may still miscalibrate, predicting risks inaccurately in certain subgroups. To improve future models, a more comprehensive approach to evaluating calibration and discrimination is needed. Calibration slopes, plots, and recalibration techniques should be used alongside discrimination metrics to provide a balanced assessment of a model's predictive accuracy.

We highlight two studies [46, 88] that used rigorous methods and transparent reporting in Boxes S1 and S2. External validation of these models in different populations is needed to determine real‐world performance and impact.

### 4.3 Implications for Research and Clinical Practice

The increasing availability of “big data” from EHR records, glucose sensors, and other sources (such as “omics studies”) has led to the development of a large number of prognostic models for GDM outcomes. Unfortunately, the lack of external validation of these models results in considerable research waste and limits the uptake of models in practice. Instead of continually developing new models, it is imperative to focus on the evaluation of existing models in new datasets. Poor performance in external validation does not necessarily indicate the need for an entirely new model. Updating or recalibrating the existing model based on both development and validation data can improve its performance, stability, and generalizability.

When the development of a new model is warranted, rigorous methodology should be followed using existing guidelines for prognosis research. Methodological guidance is available for all aspects of model development including sample size calculation, selection of predictors, shrinkage techniques, model evaluation, and internal and external validation [1, 2, 3]. Models should be reported adequately using the TRIPOD guidelines to ensure transparency and reproducibility [117].

Finally, impact studies are critical to understanding a model's true clinical value. By evaluating how a model affects clinical decision‐making, patient outcomes, resource utilization, and cost‐effectiveness, impact studies provide insight into the practical benefits of these tools [118]. Such studies are crucial for determining whether a model delivers measurable improvements in care, guiding the integration of prognostic models into practice confidently. These approaches collectively support the translation of GDM prognostic models into tools that can reliably enhance patient care and clinical efficiency.

### 4.4 Strengths and Limitations

This review demonstrates several notable strengths, including a prospective protocol registration, an extensive search across multiple databases without limitations on language or publication date and data extraction and critical appraisal using specialized tools such as CHARMS [17] and PROBAST [21]. Additionally, although nonexhaustive, the gray literature search increased the review's comprehensiveness and reduced potential publication bias. However, we were unable to assess for reporting bias as only one included study followed a prospectively published protocol. This further drives the point of the need for more transparent reporting in predictive modeling studies. An important limitation is that we were unable to compare prediction models quantitatively due to the heterogeneity of studies included. For example, GDM was diagnosed using eight different criteria and management protocols differed by center. Focusing on external validation can support future head‐to‐head comparisons of prognostic models.

## 5 Conclusion

In conclusion, this systematic review provides a comprehensive summary of current prediction models for GDM outcomes and establishes a benchmark for future model development. By identifying key methodological best practices and emphasizing the need for improved geographic representation, this review highlights critical areas for enhancement in the field. Adopting these practices—such as rigorous validation, tailored sample size calculations, and broader regional applicability—will be essential for improving the reliability, generalizability, and clinical utility of future models, ultimately supporting more effective, patient‐centered care in diverse global settings.

## Author Contributions

YAG and JEH conceived of the study. YAG was the lead reviewer for the abstract/title screening, full text screening, data extraction, and bias assessment and led the writing of both the protocol and manuscript. AK was the second reviewer for the abstract/title screening and full text screening. BO, AK, and TYL were second reviewers for the data extraction and bias assessment verification. JEH, HYL, NMA, and LM provided critical feedback and helped shape the research, analysis, and manuscript. YAG is the guarantor of this work and, as such, had full access to all the data in the study and takes responsibility for the integrity of the data and the accuracy of the data analysis.

## Conflicts of Interest

LM is a part time employee of EMIS Group plc and an advisor and shareholder of Malama Inc. The other authors declare that they have no competing interests.

## Supporting information


**Table S1.** Example Search Strategies in Medline.
**Table S2.** Framing the review eligibility criteria with PICOTS.
**Table S3.** Characteristics of the models included in the systematic review.
**Table S4.** Critical appraisal and applicability assessment of included models.
**Box S1.** Prediction model for adverse pregnancy outcomes.
**Box S2.** Prediction model for type 2 diabetic complications.
