# Supplementary material for: Prediction Models for Maternal and Offspring Short‐ and Long‐Term Outcomes Following Gestational Diabetes: A Systematic Review
Source: Obes Rev. 2025 May 4;26(9):e13934. doi: 10.1111/obr.13934 (PMC12318903; doi:10.1111/obr.13934)
Supplement: Supplementary file 1 — Table S1. Example Search Strategies in Medline. Table S2. Framing the review eligibility criteria with PICOTS. Table S3. Characteristics of the models included in the systematic review. Table S4. Critical appraisal and applicability assessment of included models. Box S1. Prediction model for adverse pregnancy outcomes. Box S2. Prediction model for type 2 diabetic complications. [file OBR-26-e13934-s001.pdf]

Full title

Prediction models for maternal and offspring short- and longer-term outcomes following gestational diabetes: a systematic review

Authors

Yasmina Al Ghadban[1](MPhil), Nerys M. Astbury[2,3] (PhD), Abdallah Kurdi[4] (BS), Ankita Sharma[1] (MSt), Beatrice Ope[5,6] (MSc), Tzu-Ying Liu[7] (MPhil), Lucy MacKillop[1,8](PhD), Huiqi Y. Lu[9] (PhD), Jane E. Hirst[1,6,8] (PhD)

Affiliations

[1]University of Oxford, Nuffield Department of Women's and Reproductive Health
[2]University of Oxford, Nuffield Department of Primary Care Health Sciences, Radcliffe Observatory Quarter, Oxford, UK
[3]NIHR Oxford Biomedical Research Centre, Oxford University Hospitals, NHS Foundation Trust, Oxford, UK
[4]Department of Biochemistry and Molecular Genetics, Faculty of Medicine, American University of Beirut, Beirut, Lebanon.
[5]Imperial College London, Faculty of Medicine, School of Public Health, London, UK
[6]The George Institute for Global Health, Imperial College, UK
[7]University of Oxford, Nuffield Department of Population Health
[8]Oxford University Hospitals NHS Foundation Trust, Oxford, UK
[9]Institute of Biomedical Engineering, Department of Engineering Science, University of Oxford

Corresponding author contact information

Yasmina Al Ghadban
Email: yasmina.alghadban@wrh.ox.ac.uk

Supplementary Table 1. Example Search Strategies in Medline

String No	Searches	Count
1	exp *Pregnancy/ or exp *Obstetrics/ or (gestation* or parturition or ante natal or antenatal* or pre natal* or prenatal* or puerper* or postnatal* or postpartum or post partum or post natal* or peripartum or peri partum or prepregnancy or pre pregnancy or preconception* or pre conception* or periconception* or peri conception* or labor or labour or birth or childbirth or newborn* or new born* or fetal or foetal or fetus or foetus or pregnancy or pregnancies or pregnant).ti,kf.	822734
2	(diabet* OR GDM OR hyperglyc* OR "glucose intolerance" OR "impaired glucose regulation" OR IGR OR "impaired fasting glucose" OR IFG OR "impaired glucose tolerance" OR IGT OR "insulin resistance").mp.	939705
3	((predict* adj5 (outcome* or risk* or model*)) or ((history or variable* or criteria or scor* or characteristic* or finding* or factor*) adj5 (predict* or model* or decision* or identif* or prognos*)) or (decision* adj3 (model* or clinical*)) or (prognostic adj5 (history or variable* or criteria or scor* or characteristic* or finding* or factor* or model*)) or (validat* or rule*)).mp. or predict*.ti.	2401291
**4**	**(1 ADJ3 2) AND 3**	**2752**
**5**	**4 NOT (exp animals/ NOT humans/)**	**2725**

Supplementary Table 2. Framing the review eligibility criteria with PICOTS

PICOTS	
Type of Model	1. Prognosis model studies without external validation in independent dataset<br>2. Prognosis model studies with external validation in independent dataset<br>3. External model validation studies with or without model updating
**P**opulation	Women with GDM as defined in the included studies, and children born to mothers with GDM
**I**ntervention	Prognostic model developed to predict maternal and child GDM outcomes
**C**omparator	Not applicable
**O**utcome	Maternal and child outcomes, listed in **Figure 1**, were selected based on outcomes studied in the literature [18] and a core outcome set developed for GDM [19]. Due to variations in outcome definitions, outcomes were collected as reported and defined in the included studies.
**T**iming	Models predicting pregnancy or birth outcomes will evaluate endpoints during the period between diagnosis with GDM until the end of the postnatal period (6 weeks after delivery). Models predicting long-term outcomes will evaluate endpoints at defined months or years after delivery.
**S**etting	The intended use of prognostic models is to perform risk stratification in the assessment of pregnant women to inform healthcare professionals' decision making and resource allocation. As such, the models are designed for use in clinical settings.

Supplementary Table 3. Characteristics of the models included in the systematic review

Author, Year	Outcome	Modelling method	Sample size (events)	No predictors		EPV	Selection of candidate predictors	Selection of final predictors	Predictors	Number (%) and handling of missing data	Type of validation	Performance measures
				Cand	Final							
Allalou (a), 2016	Type 2 Diabetes diagnosed based on American Diabetes Association criteria	Machine learning techniques (Decision Tree)	244 (122)	22	4	5.5	Based on univariable associations	Decision Trees	Metabolites [PCaeC40:5, BCAA, hexose, SM (OH) C14:1]	n (%): Unkown<br>Method: Complete-case analysis	Int: Random split data<br>Ext : None	Cal: Not evaluated<br>Disc : C-Statistic / AUC graph<br>Ov : Not evaluated<br>Util: Not evaluated
Allalou (b), 2016	Type 2 Diabetes diagnosed based on American Diabetes Association criteria	Machine learning techniques (Decision Tree)	244 (122)	2	2	61.0	Based on univariable associations	NI	2hPG, FPG	n (%): Unkown<br>Method: Complete-case analysis	Int: Random split data<br>Ext : None	Cal: Not evaluated<br>Disc : C-Statistic<br>Ov : Not evaluated<br>Util: Not evaluated
Allalou (c), 2016	Type 2 Diabetes diagnosed based on American Diabetes Association criteria	Machine learning techniques (Naïve Bayes Classifier)	244 (122)	6	6	20.3	Based on prior knowledge	Decision Trees	2hPG, FPG + Metabolites [PCaeC40:5, BCAA, hexose, SM (OH) C14:1]	n (%): Unkown<br>Method: Complete-case analysis	Int: Random split data<br>Ext : None	Cal: Not evaluated<br>Disc : C-Statistic<br>Ov : Not evaluated<br>Util: Not evaluated
Badr (a), 2022	Need for pharmacological treatment according to institutional protocol	Other	399 (77)	31	12	2.5	Based on univariable associations	Pre-specified model (not selection)	Pre-gestational weight; BMI; Multiparity; History of GDM; History of insulin therapy; Family history of DM; First-degree family history of DM; FPG; 1-h OGTT; 2-h OGTT; 2 or 3 abnormal OGTT values; HbA1c	n (%): 0 (0.0)<br>Method: Complete-case analysis	Int: None (Apparent performance)<br>Ext : Temporal	Cal: Not evaluated<br>Disc : C-Statistic / AUC graph<br>Ov : Not evaluated<br>Util: Not evaluated
Badr (b), 2022	Need for pharmacological treatment according to institutional protocol	Other	186 (76)	26	6	2.9	Based on univariable associations	Pre-specified model (not selection)	Pre-gestational weight; Pre-gestational BMI; Personal history of GDM; Personal history of insulin therapy; FPG; HbA1c	n (%): 0 (0.0)<br>Method: Complete-case analysis	Int: None (Apparent performance)<br>Ext : Temporal	Cal: Not evaluated<br>Disc : C-Statistic / AUC graph<br>Ov : Not evaluated<br>Util: Not evaluated
Badr (c), 2022	Need for pharmacological treatment according to institutional protocol	Other	375	NI	12	NI	NA	NA	Pre-gestational weight; Pre-gestational BMI; Multiparity; Personal history of GDM; Personal history of insulin therapy; Family history of DM; First-degree family history of DM; FPG; 1-h OGTT; 2-h OGTT; 2 or 3 abnormal OGTT values; HbA1c	n (%): 84 (22.4)<br>Method: No information	Int: NA<br>Ext : NA	Cal: Not evaluated<br>Disc : C-Statistic / AUC graph<br>Ov : Not evaluated<br>Util: Not evaluated

Author, Year	Outcome	Modelling method	Sample size (events)	No predictors		EPV	Selection of candidate predictors	Selection of final predictors	Predictors	Number (%) and handling of missing data	Type of validation	Performance measures
				Cand	Final							
Badr (d), 2022	Need for pharmacological treatment according to institutional protocol	Other	186	NI	6	NI	NA	NA	Pre-gestational weight; Pre-gestational BMI; Personal history of GDM; Personal history of insulin therapy; FPG; HbA1c	n (%): 84 (45.2) Method: No information	Int: NA Ext : NA	Cal: Not evaluated Disc : C-Statistic / AUC graph Ov : Not evaluated Util: Not evaluated
Barden, 2013	Type 2 diabetes ascertained from medical records and measurement of fasting glucose (>= 7 mmol/l)	Machine learning techniques (Cluster Analysis)	150 (20)	6	6	3.3	Based on prior knowledge	Decision Trees	Body mass index; Fasting Glucose; Fasting Insulin; Fasting Triglycerides; HDL cholesterol; Systolic blood pressure	n (%): Unkown Method: No information	Int: None (Apparent performance) Ext : None	Cal: Not evaluated Disc : Not evaluated Ov : Not evaluated Util: Not evaluated
Barnes (a), 2016	Need for pharmacological treatment according to institutional protocol	Logistic regression	4,015 (1302)	14	7	93.0	Based on univariable associations	Backward elimination	Maternal age; family history of diabetes; pre-pregnancy obesity; prior GDM; early diagnosis of GDM; fasting venous blood glucose level; HbA1c at GDM diagnosis	n (%): 698 (17.4) Method: No information	Int: None (Apparent performance) Ext : Different setting	Cal: Not evaluated Disc : C-Statistic Ov : Not evaluated Util: Not evaluated
Barnes (b), 2016	NI	Logistic regression	1,040 (491)	NI	7	NI	NA	NA	Maternal age; Family history of diabetes; pre-pregnancy obesity; Prior GDM; Early diagnosis of GDM; Fasting venous blood glucose level; HbA1c at GDM diagnosis	n (%): Unkown Method: No information	Int: NA Ext : NA	Cal: Not evaluated Disc : C-Statistic Ov : Not evaluated Util: Not evaluated
Bartakova, 2021	Persistent postpartum glucose intolerance based on the WHO criteria for non-pregnant subjects	Logistic regression	244 (22)	28	5	0.8	Based on univariable associations	Backward elimination	FPG in mid-trimester OGTT; obesity; family history of diabetes; instrumental delivery; personal history of hypothyroidism	n (%): 57 (23.4) Method: No information	Int: None (Apparent performance) Ext : None	Cal: Not evaluated Disc : C-Statistic / AUC graph Ov : Not evaluated Util: Not evaluated
Bengtson, 2022	Impaired glucose tolerance diagnosed with HbA1c >= 5.7% at 1-year postpartum	Logistic regression	203 (71)	31	8	2.3	Based on univariable associations	LASSO selection	Prepregnancy weight; BMI prepregnancy; BMI prepregnancy; previous GDM gravidity; family history of diabetes; GDM diagnosis <24wkGA; FPG 2 days postpartum; 2h plasma glucose 2 days postpartum	n (%): Unkown Method: Multiple imputation	Int: Cross-validation Ext : None	Cal: Calibration plot Disc : C-Statistic Ov : Not evaluated Util: Not evaluated
				18	12	26.4						

Author, Year	Outcome	Modelling method	Sample size (events)	No predictors		EPV	Selection of candidate predictors	Selection of final predictors	Predictors	Number (%) and handling of missing data	Type of validation	Performance measures
				Cand	Final							
Cooray (a), 2022	Maternal and perinatal complications (Hypertensive disorders of pregnancy, large-for-gestational age neonate, neonatal hypoglycaemia requiring intravenous therapy, shoulder dystocia, perinatal death, neonatal bone fracture and nerve palsy)	Logistic regression	1,747 (476)				Based on prior knowledge	LASSO selection	Maternal age; Pre-pregnancy BMI; Fasting glucose OGTT; 1-hour glucose OGTT; Gestation at GDM diagnosis; Southern and Central Asian; East Asian; Nulliparity; Previous pre-eclampsia; Gestational weight gain to GDM diagnosis per week; Family history of diabetes; Previous LGA baby	n (%): Unkown<br>Method: Multiple imputation	Int: Bootstrap<br>Ext : Temporal	Cal: Calibration plot / Slope / CITL / E:O ratio<br>Disc : C-Statistic<br>Ov : Not evaluated<br>Util: DCA
Cooray (b), 2022	As above	Logistic regression	955 (244)	NI	12	NI	NA	NA	As above	n (%): Unkown<br>Method: Multiple imputation	Int: NA<br>Ext : NA	Cal: Calibration plot / Slope / CITL / E:O ratio<br>Disc : C-Statistic / AUC graph<br>Ov : Not evaluated<br>Util: DCA
Cormier (a), 2014	Pre-diabetes diagnosed based on the 2013 Canadian Diabetes Association guidelines	Logistic Regression	214 (135)	NI	NI	NI	NI	NI	NI	n (%): Unkown<br>Method: No information	Int: None (Apparent performance)<br>Ext : None	Cal: Not evaluated<br>Disc : C-Statistic / AUC graph<br>Ov : Not evaluated<br>Util: Not evaluated
Cormier (b), 2014	Type 2 Diabetes defined as fasting plasma glucose ≥7.0 mmol/l, and/or 2-hour plasma glucose post-OGTT ≥ 11.1 mmol/l, and/or A1C ≥ 6.5%.	Logistic Regression	214 (40)	NI	NI	NI	NI	NI	Unclear	n (%): Unkown<br>Method: No information	Int: None (Apparent performance)<br>Ext : None	Cal: Not evaluated<br>Disc : C-Statistic / AUC graph<br>Ov : Not evaluated<br>Util: Not evaluated
Du, 2021	Need for insulin according to institutional protocol	Cox regression	626 (188)	19	7	9.9	Based on prior knowledge	LASSO selection	Age; gestational age at GDM diagnosis; BMI at GDM diagnosis; first degree family history of T2DM; GDM history; FPG; HbA1c	n (%): 220 (35.1)<br>Method: Complete-case analysis	Int: Bootstrap<br>Ext : None	Cal: Calibration plot<br>Disc : C-Statistic<br>Ov : Not evaluated<br>Util: DCA
				8	8	16.3						

Author, Year	Outcome	Modelling method	Sample size (events)	No predictors		EPV	Selection of candidate predictors	Selection of final predictors	Predictors	Number (%) and handling of missing data	Type of validation	Performance measures
				Cand	Final							
Eleftheriades (a), 2021	Need for insulin according to institutional protocol	Machine learning techniques (CART)	775 (130)				Based on prior knowledge	Backward elimination	Maternal age; smoking; method of conception; maternal BMI before conception; blood glucose levels at baseline; blood glucose levels at 1 h; blood glucose levels at 2 h during the OGTT; and z scores for the fetal abdominal circumference in the second trimester	n (%): Unkown<br>Method: No information	Int: Cross-validation<br>Ext : Different setting	Cal: Not evaluated<br>Disc : C-Statistic<br>Ov : Not evaluated<br>Util: Not evaluated
Eleftheriades (b), 2021	Need for insulin according to institutional protocol	Machine learning techniques (CART)	168 (28)	NI	8	NI	NA	NA	As above	n (%): Unkown<br>Method: No information	Int: NA<br>Ext : NA	Cal: Not evaluated<br>Disc : C-Statistic<br>Ov : Not evaluated<br>Util: Not evaluated
Elnour, 2006	Postpartum diabetes diagnosed based on the Expert Committee on the Diagnosis and Classification of Diabetes Mellitus criteria	Logistic regression	165 (34)	18	4	1.9	Based on univariable associations	Backward elimination	Severe hyperglycaemia; Family history of diabetes mellitus; Gravidity; Gestational age at diagnosis	n (%): Unkown<br>Method: No information	Int: None (Apparent performance)<br>Ext : None	Cal: Calibration plot / HL test<br>Disc : C-Statistic / AUC graph<br>Util: Not evaluated
Ford, 2022	Need for insulin according to institutional protocol	Logistic regression	2,048 (647)	11	12	58.8	Based on prior knowledge	Backward elimination	Age; BMI; ethnicity previous GDM; previous birthweight > 90%; gestational age at OGTT; OGTT fasting result; OGTT 1hr result; OGTT 2hr result	n (%): 62 (3.0)<br>Method: Multiple imputation	Int: Cross-validation<br>Ext : None	Cal: Calibration plot / Slope / CITL / E:O ratio<br>Disc : C-Statistic / AUC graph<br>Ov : Not evaluated<br>Util: Not evaluated
Hahn, 2023	Recurrent GDM (GDM in the pregnancy following the index pregnancy with GDM)	Logistic Regression	159 (115)	8	3	5.5	Based on univariable associations	Other	Family history; overweight (in subsequent pregnancy); caesarean section (in index pregnancy)	n (%): Unkown<br>Method: No Information	Int: None (Apparent performance)<br>Ext : None	Cal: Not evaluated<br>Disc : C-Statistic<br>Ov : Not evaluated<br>Util: Not evaluated
Harper, 2016	Glyburide failure was defined as reaching glyburide 20 mg/day and receiving insulin. Glyburide success was defined as any glyburide dose without insulin and > 70% of visits with glycemic control.	Logistic regression	220 (67)	23	6	2.9	Based on univariable associations	Backward elimination	GDM in prior pregnancy; Gestational age at diagnosis; 1h glucose challenge test; Blood sugar measurements	n (%): Unkown<br>Method: No information	Int: Bootstrap<br>Ext : None	Cal: Not evaluated<br>Disc : C-Statistic / AUC graph<br>Ov : Not evaluated<br>Util: Not evaluated
			564 (71)	13	4	5.5						

Author, Year	Outcome	Modelling method	Sample size (events)	No predictors		EPV	Selection of candidate predictors	Selection of final predictors	Predictors	Number (%) and handling of missing data	Type of validation	Performance measures
				Cand	Final							
Huang (a), 2023	Preterm delivery <37 weeks' gestation	Logistic Regression					Based on univariable associations	Pre-specified model (not selection)	Maternal age; Insulin use; neutrophil-lymphocyte ratio; monocyte count	n (%): Unkown<br>Method: No Information	Int: Bootstrap<br>Ext : Temporal	Cal: Calibration plot / HL test<br>Disc : C-Statistic / AUC graph<br>Util: DCA
Huang (b), 2023	Preterm delivery <37 weeks' gestation	Logistic Regression	242 (32)	NI	4	NI	NA	NA	As above	n (%): Unkown<br>Method: No Information	Int: NA<br>Ext : NA	Cal: Calibration plot / HL test<br>Disc : C-Statistic / AUC graph<br>Ov : Not evaluated<br>Util: DCA
Ignell (a), 2016	Postpartum diabetes diagnosed based on WHO 1999 criteria	Logistic regression	200 (67)	16	3	4.2	Based on univariable associations	Backward elimination	European ethnicity; 2hr glucose concentration during pregnancy; BMI from the 1- to 2-year follow-up	n (%): Unkown<br>Method: No information	Int: None (Apparent performance)<br>Ext : None	Cal: Not evaluated<br>Disc : C-Statistic<br>Ov : Not evaluated<br>Util: Not evaluated
Ignell (b), 2016	Postpartum diabetes diagnosed based on WHO 1999 criteria	Logistic regression	64 (28)	16	3	1.8	Based on univariable associations	Forward selection	Age at delivery; 2hr glucose concentration during pregnancy; BMI from the 1- to 2-year follow-up	n (%): Unkown<br>Method: No information	Int: None (Apparent performance)<br>Ext : None	Cal: Not evaluated<br>Disc : C-Statistic<br>Ov : Not evaluated<br>Util: Not evaluated
Ingram (a), 2017	Return for OGTT at 6-12 weeks postpartum	Other	165 (48)	16	4	3.0	Based on prior knowledge	Backward elimination	Parity; Smoker during pregnancy; BMI; pregnancy OGTT results	n (%): 16 (9.7)<br>Method: Single imputation	Int: None (Apparent performance)<br>Ext : None	Cal: Not evaluated<br>Disc : C-Statistic<br>Ov : Not evaluated<br>Util: Not evaluated
Ingram (b), 2017	Abnormal result on postpartum OGTT consisting of impaired fasting glucose, impaired glucose tolerance and diabetes at 6-12 weeks postpartum. Impaired fasting glucose, IGT and diabetes were combined to create a composite variable coded as abnormal for the primary outcome measure.	Other	117 (23)	16	4	1.4	Based on prior knowledge	Backward elimination	High risk ethnicity; induction of labour; past history of GDM; pregnancy OGTT results	n (%): 16 (13.7)<br>Method: Single imputation	Int: None (Apparent performance)<br>Ext : None	Cal: Not evaluated<br>Disc : C-Statistic<br>Ov : Not evaluated<br>Util: Not evaluated
Ingram (c), 2017	As above	Other	148 (37)	16	4	2.3	Based on prior knowledge	Backward elimination	High risk ethnicity; induction of labour; past history of GDM; pregnancy OGTT results	n (%): 16 (10.8)<br>Method: Single imputation	Int: None (Apparent performance)<br>Ext : None	Cal: Not evaluated<br>Disc : C-Statistic<br>Ov : Not evaluated<br>Util: Not evaluated

Author, Year	Outcome	Modelling method	Sample size (events)	No predictors		EPV	Selection of candidate predictors	Selection of final predictors	Predictors	Number (%) and handling of missing data	Type of validation	Performance measures
				Cand	Final							
Joglekar (a), 2021	Type 2 Diabetes diagnosed based on American Diabetes Association criteria	NI	82 (11)	10	6	1.1	Based on univariable associations	Pre-specified model (not selection)	Age; BMI; Pregnancy fasting glucose; Postnatal fasting glucose; Cholesterol; Triacylglycerol;	n (%): Unkown<br>Method: Complete-case analysis	Int: None (Apparent performance)<br>Ext : None	Cal: Not evaluated<br>Disc : C-Statistic<br>Ov : Not evaluated<br>Util: Not evaluated
Joglekar (b), 2021	Type 2 Diabetes diagnosed based on American Diabetes Association criteria	NI	82 (11)	10	7	1.1	Based on univariable associations	Pre-specified model (not selection)	As above	n (%): Unkown<br>Method: Complete-case analysis	Int: None (Apparent performance)<br>Ext : None	Cal: Not evaluated<br>Disc : C-Statistic<br>Ov : Not evaluated<br>Util: Not evaluated
Jotic, 2023	Postpartum dyslipidemia diagnosed according according to the Third Report of the National Cholesterol Education Program (NCEP) Expert Panel on Detection, Evaluation, and Treatment of High Blood Cholesterol in Adults (Adult Treatment Panel III)	Logistic Regression	147 (63)	11	6	5.7	Based on univariable associations	Pre-specified model (not selection)	Age; preconception BMI; HbA1c; FPG; LDL; triglycerides	n (%): Unkown<br>Method: No Information	Int: None (Apparent performance)<br>Ext : None	Cal: Not evaluated<br>Disc : C-Statistic / AUC graph<br>Ov : Not evaluated<br>Util: Not evaluated
Kang (a), 2019	Macrosomia defined as a birth weight ≥ 4000g	Machine learning techniques (Neural network)	1,891 (268)	NI	10	NI	Based on univariable associations	Pre-specified model (not selection)	Antenatal uterine height; prenatal BMI; abdominal girth; HbA1c; HDL-c; gestational weeks; TG; ketone body; family history of diabetes; times of antenatal examination	n (%): Unkown<br>Method: Complete-case analysis	Int: Random split data<br>Ext : None	Cal: Not evaluated<br>Disc : AUC graph<br>Ov : Not evaluated<br>Util: Not evaluated
Kang (b), 2019	Macrosomia defined as a birth weight ≥4000g	Machine learning techniques (Neural network)	1,891 (268)	NI	7	NI	Based on univariable associations	Stepwise selection	Antenatal uterine height; abdominal girth; HDL-c; gestational weeks; family history of diabetes; times of antenatal examination; prenatal BMI	n (%): Unkown<br>Method: Complete-case analysis	Int: Random split data<br>Ext : None	Cal: Not evaluated<br>Disc : AUC graph<br>Ov : Not evaluated<br>Util: Not evaluated
Khan, 2019	Type 2 Diabetes diagnosed based on American Diabetes Association criteria	Machine learning techniques (Filtered Classifier Algorithm)	140 (55)	75	7	0.7	Based on univariable associations	No NI	CE(16:0); NEFA(22:4); TAG 48:2 FA 16:1; CE(20:4); PE(P-18:0/18:1); TAG 54:0 FA 16:0; TAG 50:1 FA 16:0	n (%): Unkown<br>Method: Complete-case analysis	Int: Cross-validation<br>Ext : None	Cal: Not evaluated<br>Disc : C-Statistic / AUC graph<br>Ov : Not evaluated<br>Util: Not evaluated
Kim, 2022			660 (77)	18	3	4.3						

Author, Year	Outcome	Modelling method	Sample size (events)	No predictors		EPV	Selection of candidate predictors	Selection of final predictors	Predictors	Number (%) and handling of missing data	Type of validation	Performance measures
				Cand	Final							
	Large for Gestational Age defined as having a birth weight of ≥90%.	Logistic regression					Based on prior knowledge	Backward elimination	Head circumference measured at GDM screening Z-score; Abdominal circumference within 2 weeks before delivery Z-score; Pre-pregnancy BMI	n (%): Unkown<br>Method: No information	Int: Cross-validation<br>Ext : None	Cal: Not evaluated<br>Disc : C-Statistic / AUC graph<br>Ov : Not evaluated<br>Util: Not evaluated
Koefoed, 2023	Pharmacological treatment according to Danish Guidelines	Logistic Regression	1,104 (282)	12	7	23.5	Based on univariable associations	Pre-specified model (not selection)	Family history of diabetes; Current smoker; Parity; Prepregnancy BMI; gestational age at OGTT; 2-h OGTT result; HbA1c at diagnosis	n (%): Unkown<br>Method: Complete-case analysis	Int: None (Apparent performance)<br>Ext : None	Cal: Not evaluated<br>Disc : C-Statistic / AUC graph<br>Ov : Not evaluated<br>Util: Not evaluated
Kohler, 2016	Postpartum diabetes diagnosed based on American Diabetes Association criteria	Cox regression	304 (147)	6	4	24.5	Based on prior knowledge	LASSO selection	BMI in early pregnancy; Insulin treatment; Family history of diabetes; Lactation	n (%): 47 (15.5)<br>Method: Complete-case analysis	Int: Random split data<br>Ext : None	Cal: Slope<br>Disc : C-Statistic / Risk group curves<br>Ov : R-squared<br>Util: Not evaluated
Kondo (a), 2018	Postpartum diabetes diagnosed based on WHO 1999 criteria	Logistic regression	123 (45)	18	4	2.5	Based on univariable associations	Pre-specified model (not selection)	Age; family history of diabetes; BMI; use of insulin during pregnancy	n (%): Unkown<br>Method: No information	Int: Bootstrap<br>Ext : None	Cal: HL test<br>Disc : C-Statistic<br>Ov : Not evaluated<br>Util: DCA
Kondo (b), 2018	Postpartum diabetes diagnosed based on WHO 1999 criteria	Logistic regression	123 (45)	18	5	2.5	Based on univariable associations	Pre-specified model (not selection)	Age; family history of diabetes; BMI; use of insulin during pregnancy; insulinogenic index/fasting immunoreactive insulin	n (%): Unkown<br>Method: No information	Int: Bootstrap<br>Ext : None	Cal: Calibration plot / HL test<br>Disc : C-Statistic<br>Ov : Not evaluated<br>Util: DCA
Kondo (c), 2018	Postpartum diabetes diagnosed based on WHO 1999 criteria	Logistic regression	123 (45)	18	5	2.5	Based on univariable associations	Pre-specified model (not selection)	Age; family history of diabetes; BMI; use of insulin during pregnancy; total glucose	n (%): Unkown<br>Method: No information	Int: Bootstrap<br>Ext : None	Cal: Calibration plot / HL test<br>Disc : C-Statistic<br>Ov : Not evaluated<br>Util: DCA
Kwak (a), 2013	Type 2 diabetes diagnosed based on American Diabetes Association criteria	Logistic regression	395 (116)	NI	3	NI	NI	NI	Age; family history of diabetes; prepregnancy BMI	n (%): Unkown<br>Method: No information	Int: None (Apparent performance)<br>Ext : None	Cal: HL test<br>Disc : C-Statistic / AUC graph<br>Ov : Not evaluated<br>Util: Not evaluated
Kwak (b), 2013	Type 2 diabetes diagnosed based on American Diabetes Association criteria	Logistic regression	395 (116)	NI	4	NI	NI	NI	Age; family history of diabetes; prepregnancy BMI; wGRS	n (%): Unkown<br>Method: No information	Int: None (Apparent performance)<br>Ext : None	Cal: HL test<br>Disc : C-Statistic / AUC graph<br>Ov : Not evaluated<br>Util: Not evaluated
				NI	6	NI	NI	NI				

Author, Year	Outcome	Modelling method	Sample size (events)	No predictors		EPV	Selection of candidate predictors	Selection of final predictors	Predictors	Number (%) and handling of missing data	Type of validation	Performance measures
				Cand	Final							
Kwak (c), 2013	Type 2 diabetes diagnosed based on American Diabetes Association criteria	Logistic regression	395 (116)						Age; family history of diabetes; prepregnancy BMI; mean arterial pressure; fasting glucose; fasting insulin concentrations	n (%): Unkown<br>Method: No information	Int: None (Apparent performance)<br>Ext : None	Cal: HL test<br>Disc : C-Statistic / AUC graph<br>Ov : Not evaluated<br>Util: Not evaluated
Kwak (d), 2013	Type 2 diabetes diagnosed based on American Diabetes Association criteria	Logistic regression	395 (116)	NI	7	NI	NI	NI	Age, family history of diabetes; prepregnancy BMI; mean arterial pressure; fasting glucose; fasting insulin concentrations; wGRS	n (%): Unkown<br>Method: No information	Int: None (Apparent performance)<br>Ext : None	Cal: HL test<br>Disc : C-Statistic / AUC graph<br>Ov : Not evaluated<br>Util: Not evaluated
Lai, 2020	Type 2 diabetes diagnosed based on American Diabetes Association criteria	Machine learning techniques (Random Forest)	337 (98)	30	20	3.3	Based on univariable associations	Bootstrap selection	Metabolic signature (hexose; his; spermidine; AC10; kynurenine; lysoPC a C26:0; total DMA; PC ae C40:4; Ser; Glu; PC aa C30:0; Ile; AC3; lyso PC a C26:1; SM (OH) C22:2; Gln; PC aa C32:1; SM C20:2)	n (%): Unkown<br>Method: No information	Int: Random split data<br>Ext : None	Cal: Not evaluated<br>Disc : C-Statistic / AUC graph<br>Ov : Not evaluated<br>Util: Not evaluated
Lappas (a), 2015	Type 2 diabetes diagnosed based on American Diabetes Association criteria	Logistic regression	104 (21)	6	6	3.5	Based on prior knowledge	Pre-specified model (not selection)	Age; BMI; pregnancy fasting glucose; postnatal fasting glucose; triacylglycerol and total cholesterol	n (%): Unkown<br>Method: Complete-case analysis	Int: Cross-validation<br>Ext : None	Cal: Not evaluated<br>Disc : C-Statistic / AUC graph<br>Ov : Not evaluated<br>Util: Not evaluated
Lappas (b), 2015	Type 2 diabetes diagnosed based on American Diabetes Association criteria	Logistic regression	104 (21)	11	3	1.9	Based on univariable associations	Stepwise selection	CE 20:4; PE(P-36:2) and PS 38:4	n (%): Unkown<br>Method: Complete-case analysis	Int: Cross-validation<br>Ext : None	Cal: Not evaluated<br>Disc : C-Statistic / AUC graph<br>Ov : Not evaluated<br>Util: Not evaluated
Lappas (c), 2015	Type 2 diabetes diagnosed based on American Diabetes Association criteria	Logistic regression	104 (21)	19	9	1.1	Based on univariable associations	Stepwise selection	Age; BMI; pregnancy fasting glucose; postnatal fasting glucose; triacylglycerol; total cholesterol; CE 20:4; PE(P-36:2) and PS 38:4	n (%): Unkown<br>Method: Complete-case analysis	Int: Cross-validation<br>Ext : None	Cal: Not evaluated<br>Disc : C-Statistic / AUC graph<br>Ov : Not evaluated<br>Util: Not evaluated
Lappas (a), 2016	Type 2 diabetes diagnosed based on American Diabetes Association criteria	Logistic regression	95 (21)	4	4	5.3	Based on prior knowledge	Pre-specified model (not selection)	Age; BMI; pregnancy fasting glucose; postnatal fasting glucose	n (%): Unkown<br>Method: Complete-case analysis	Int: None (Apparent performance)<br>Ext : None	Cal: Not evaluated<br>Disc : C-Statistic / AUC graph<br>Ov : Not evaluated<br>Util: Not evaluated
Lappas (b), 2016	Type 2 diabetes diagnosed based on American Diabetes Association criteria	Logistic regression	95 (21)	4	2	5.3	Based on univariable associations	Pre-specified model (not selection)	IGF-I; IGFBP-2	n (%): Unkown<br>Method: Complete-case analysis	Int: None (Apparent performance)<br>Ext : None	Cal: Not evaluated<br>Disc : C-Statistic / AUC graph<br>Ov : Not evaluated<br>Util: Not evaluated

Author, Year	Outcome	Modelling method	Sample size (events)	No predictors		EPV	Selection of candidate predictors	Selection of final predictors	Predictors	Number (%) and handling of missing data	Type of validation	Performance measures
				Cand	Final							
Lappas (c), 2016	Type 2 diabetes diagnosed based on American Diabetes Association criteria	Logistic regression	95 (21)	8	6	2.6	Based on univariable associations	Pre-specified model (not selection)	Age; BMI; pregnancy fasting glucose; postnatal fasting glucose; IGF-I; IGFBP-2	n (%): Unkown Method: Complete-case analysis	Int: None (Apparent performance) Ext : None	Cal: Not evaluated Disc : C-Statistic / AUC graph Ov : Not evaluated Util: Not evaluated
Lappas (a), 2018	Type 2 diabetes diagnosed based on American Diabetes Association criteria	Logistic regression	98 (20)	4	4	5.0	Based on prior knowledge	Pre-specified model (not selection)	Age; BMI; pregnancy fasting glucose; postnatal fasting glucose	n (%): Unkown Method: Complete-case analysis	Int: None (Apparent performance) Ext : None	Cal: Not evaluated Disc : C-Statistic / AUC graph Ov : Not evaluated Util: Not evaluated
Lappas (b), 2018	Type 2 diabetes diagnosed based on American Diabetes Association criteria	Logistic regression	98 (20)	11	5	1.8	Based on univariable associations	Pre-specified model (not selection)	Apo CIII; Apo CIII/Apo AI; Apo CIII/Apo AII; Apo CIII/Apo CII; Apo CIII/Apo E	n (%): Unkown Method: Complete-case analysis	Int: None (Apparent performance) Ext : None	Cal: Not evaluated Disc : C-Statistic / AUC graph Ov : Not evaluated Util: Not evaluated
Lappas (c), 2018	Type 2 diabetes diagnosed based on American Diabetes Association criteria	Logistic regression	98 (20)	15	9	1.3	Based on univariable associations	Pre-specified model (not selection)	Age; BMI; pregnancy fasting glucose; postnatal fasting glucose; Apo CIII; Apo CIII/Apo AI; Apo CIII/Apo AII; Apo CIII/Apo CII; Apo CIII/Apo E	n (%): Unkown Method: Complete-case analysis	Int: None (Apparent performance) Ext : None	Cal: Not evaluated Disc : C-Statistic / AUC graph Ov : Not evaluated Util: Not evaluated
Liao (a), 2022	Treatment modality (MNT or pharmacological treatment) according to institutional protocol	Logistic regression	27,240 (10289)	68	3	151.3	Based on prior knowledge	Other	History of GDM, pre-pregnancy obesity, and prediabetes before pregnancy	n (%): Unkown Method: Multiple imputation	Int: Cross-validation Ext : Temporal	Cal: Not evaluated Disc : C-Statistic Ov : Not evaluated Util: Not evaluated
Liao (b), 2022	As above	Logistic regression	27,240 (10289)	94	4	109.5	Based on prior knowledge	Other	History of GDM; pre-pregnancy obesity; glucose levels at 50-g, 1-h glucose challenge test for GDM screening; prediabetes before pregnancy	n (%): Unkown Method: Multiple imputation	Int: Cross-validation Ext : Temporal	Cal: Not evaluated Disc : C-Statistic Ov : Not evaluated Util: Not evaluated
Liao (c), 2022	As above	Logistic regression	27,240 (10289)	106	3	97.1	Based on prior knowledge	Other	Fasting glucose value at 100g, 3h oral glucose tolerance test; gestational week at GDM diagnosis; GDM diagnosis by Carpenter-Coustan criteria (versus by fasting hyperglycemia)	n (%): Unkown Method: Multiple imputation	Int: Cross-validation Ext : Temporal	Cal: Not evaluated Disc : C-Statistic Ov : Not evaluated Util: Not evaluated
Liao (d), 2022	As above			176	4	58.5		Other				

Author, Year	Outcome	Modelling method	Sample size (events)	No predictors		EPV	Selection of candidate predictors	Selection of final predictors	Predictors	Number (%) and handling of missing data	Type of validation	Performance measures
				Cand	Final							
		Logistic regression	27,240 (10289)				Based on prior knowledge		Gestational week at GDM diagnosis; fasting glucose value at 100-g, 3-h oral glucose tolerance test; self-monitored glycemic control status at fasting; number of fasting self-monitored blood glucose measurements	n (%): Unkown Method: Multiple imputation	Int: Cross-validation Ext : Temporal	Cal: Not evaluated Disc : C-Statistic Ov : Not evaluated Util: Not evaluated
Liao (e), 2022	As above	Logistic regression	3,234 (1528)	NA	3	NA	NA	NA	History of GDM; pre-pregnancy obesity; prediabetes before pregnancy	n (%): Unkown Method: Multiple imputation	Int: NA Ext : NA	Cal: Not evaluated Disc : C-Statistic / AUC graph Ov : Not evaluated Util: Not evaluated
Liao (f), 2022	As above	Logistic regression	3,234 (1528)	NA	4	NA	NA	NA	History of GDM; pre-pregnancy obesity; glucose levels at 50-g, 1-h glucose challenge test for GDM screening; prediabetes before pregnancy	n (%): Unkown Method: Multiple imputation	Int: NA Ext : NA	Cal: Not evaluated Disc : C-Statistic / AUC graph Ov : Not evaluated Util: Not evaluated
Liao (g), 2022	As above	Logistic regression	3,234 (1528)	NA	3	NA	NA	NA	Fasting glucose value at 100-g, 3-h oral glucose tolerance test; gestational week at GDM diagnosis; GDM diagnosis by Carpenter-Coustan criteria (versus by fasting hyperglycemia)	n (%): Unkown Method: Multiple imputation	Int: NA Ext : NA	Cal: Not evaluated Disc : C-Statistic / AUC graph Ov : Not evaluated Util: Not evaluated
Liao (h), 2022	As above	Logistic regression	3,234 (1528)	NA	4	NA	NA	NA	Gestational week at GDM diagnosis; fasting glucose value at 100-g, 3-h oral glucose tolerance test; self-monitored glycemic control status at fasting; number of fasting self-monitored blood glucose measurements	n (%): Unkown Method: Multiple imputation	Int: NA Ext : NA	Cal: Not evaluated Disc : C-Statistic / AUC graph Ov : Not evaluated Util: Not evaluated
Lin, 2011	Diabetes mellitus (diagmosed with a fasting value ≥126 mg/dl or 2-h 75-g OGTT value ≥200 mg/dl); and pre-diabetes mellitus (diagnosed with a 110 mg/dl ≤ fasting value <126 mg/dl or 140 mg/dl ≤2-h 75-g OGTT value <200 mg/ dl)	Machine learning techniques (AIRS)	152 (42)	12	NI	3.5	All available predictors	Other	Unclear	n (%): Unkown Method: No Information	Int: Cross-validation Ext : None	Cal: Not evaluated Disc : Not evaluated Ov : Not evaluated Util: Not evaluated
Li (a), 2018				5	4	16.6						

Author, Year	Outcome	Modelling method	Sample size (events)	No predictors		EPV	Selection of candidate predictors	Selection of final predictors	Predictors	Number (%) and handling of missing data	Type of validation	Performance measures
				Cand	Final							
	Type 2 diabetes diagnosed based on American Diabetes Association criteria	Cox regression	1,263 (83)				Based on prior knowledge	Backward elimination	Family history of diabetes; BMI category before pregnancy; pregnancy-induced hypertension; 2h 75g glucose category at 26-30 gestational weeks	n (%): Unkown<br>Method: No information	Int: None (Apparent performance)<br>Ext : None	Cal: Calibration plot<br>Disc : C-Statistic<br>Ov : Not evaluated<br>Util: Not evaluated
Li (b), 2018	Type 2 diabetes diagnosed based on American Diabetes Association criteria	Cox regression	1,263 (83)	5	4	16.6	Based on prior knowledge	Backward elimination	As above	n (%): Unkown<br>Method: No information	Int: None (Apparent performance)<br>Ext : None	Cal: Calibration plot<br>Disc : C-Statistic<br>Ov : Not evaluated<br>Util: Not evaluated
Lu (a), 2022	Mode of delivery (spontaneous vaginal delivery or assisted delivery vs planned or emergency caesarean)	Logistic regression	155 (35)	12	5	2.9	Based on univariable associations	Backward elimination	Maternal weight gain category; maternal height category; HbA1C category; parity category, and OGGT fasting category	n (%): Unkown<br>Method: No information	Int: None (Apparent performance)<br>Ext : None	Cal: Not evaluated<br>Disc : C-Statistic / AUC graph<br>Ov : Not evaluated<br>Util: Not evaluated
Lu (b), 2022	Mode of delivery (spontaneous vaginal delivery or assisted delivery vs planned or emergency caesarean)	Logistic regression	97 (26)	26	5	1.0	All available predictors	LASSO selection	Previous caesarean sectionl OGGT2hour; HbA1c; Fasting BG Mean3336 (mean blood glucose of all six-tag readings at or before 32 weeks of pregnancy and between 33 and 36 weeks of pregnancy); Fasting BG BBWeek1 (first week average fasting blood glucose values before the last blood glucose measurements)	n (%): Unkown<br>Method: Multiple imputation	Int: Cross-validation<br>Ext : None	Cal: Not evaluated<br>Disc : C-Statistic<br>Ov : Not evaluated<br>Util: Not evaluated
Man, 2021	Type 2 diabetes diagnosed based on American Diabetes Association criteria	Cox regression	317 (82)	11	5	7.5	Based on univariable associations	Pre-specified model (not selection)	GDM treatment method; BMI; fasting glucose; Hb1Ac; interaction between treatment and BMI	n (%): Unkown<br>Method: No information	Int: Cross-validation<br>Ext : None	Cal: Not evaluated<br>Disc : C-Statistic / AUC graph / Risk group curves<br>Ov : R-squared<br>Util: Not evaluated
Marozas (a), 2018	No informaton	Machine learning techniques (Ensemble model)	151 (85)	44	12	1.5	Based on prior knowledge	Other	Income level; sugar consumption; rs7903146_CC; rs12255372_GG; desserts; year of first given birth; weight before pregnancy with GDM; weight of first born child; physical activity; number of given births; residence; time needed to get to work	n (%): Unkown<br>Method: Other	Int: Cross-validation<br>Ext : None	Cal: Not evaluated<br>Disc : C-Statistic<br>Ov : Not evaluated<br>Util: Not evaluated
	No informaton		151 (85)	44	9	1.5		Other				

Author, Year	Outcome	Modelling method	Sample size (events)	No predictors		EPV	Selection of candidate predictors	Selection of final predictors	Predictors	Number (%) and handling of missing data	Type of validation	Performance measures
				Cand	Final							
Marozas (b), 2018		Machine learning techniques (Ensemble model)					Based on prior knowledge		Sugar consumption; income level; number of given births; education; weight of first born child; year of first given birth; weight before pregnancy with GDM; rs12255372_GT; desserts	n (%): Unkown<br>Method: Other	Int: Cross-validation<br>Ext : None	Cal: Not evaluated<br>Disc : C-Statistic<br>Ov : Not evaluated<br>Util: Not evaluated
McIntyre (a), 2018	Primary caesarean delivery	Logistic regression	1,248 (241)	7	7	34.4	All available predictors	Pre-specified model (not selection)	Fasting, one hour and two hour OGTT results; age; height; BMI; parity	n (%): Unkown<br>Method: Complete-case analysis	Int: None (Apparent performance)<br>Ext : None	Cal: Not evaluated<br>Disc : C-Statistic / AUC graph<br>Ov : Not evaluated<br>Util: Not evaluated
McIntyre (b), 2018	Birth injury including shoulder dystocia	Logistic regression	1,248 (29)	7	7	4.1	All available predictors	Pre-specified model (not selection)	Fasting, one hour and two hour OGTT results; age; height; BMI; parity	n (%): Unkown<br>Method: Complete-case analysis	Int: None (Apparent performance)<br>Ext : None	Cal: Not evaluated<br>Disc : C-Statistic / AUC graph<br>Ov : Not evaluated<br>Util: Not evaluated
McIntyre (c), 2018	Birth > 90th centile in comparison to the Australian national standard values published by Roberts and Lancaster	Logistic regression	1,248 (175)	7	7	25.0	All available predictors	Pre-specified model (not selection)	Fasting, one hour and two hour OGTT results; age; height; BMI; parity	n (%): Unkown<br>Method: Complete-case analysis	Int: None (Apparent performance)<br>Ext : None	Cal: Not evaluated<br>Disc : C-Statistic / AUC graph<br>Ov : Not evaluated<br>Util: Not evaluated
McIntyre (d), 2018	Neonatal adiposity defined as % body fat > 90th centile derived from within the Mater HAPO dataset	Logistic regression	1,000 (100)	7	7	14.3	All available predictors	Pre-specified model (not selection)	Fasting, one hour and two hour OGTT results; age; height; BMI; parity	n (%): Unkown<br>Method: Complete-case analysis	Int: None (Apparent performance)<br>Ext : None	Cal: Not evaluated<br>Disc : C-Statistic / AUC graph<br>Ov : Not evaluated<br>Util: Not evaluated
McIntyre (e), 2018	Hyperinsulinaemia defined as an elevated cord c-peptide (>1.7 μg/L, 90th centile in the HAPO dataset)	Logistic regression	992 (76)	7	7	10.9	All available predictors	Pre-specified model (not selection)	Fasting, one hour and two hour OGTT results; age; height; BMI; parity	n (%): Unkown<br>Method: Complete-case analysis	Int: None (Apparent performance)<br>Ext : None	Cal: Not evaluated<br>Disc : C-Statistic / AUC graph<br>Ov : Not evaluated<br>Util: Not evaluated
McIntyre (f), 2018	Hypoglycaemia defined as blood glucose level of <2.2 mmol/L (10th centile in the HAPO dataset)	Logistic regression	695 (73)	7	7	10.4	All available predictors	Pre-specified model (not selection)	Fasting, one hour and two hour OGTT results; age; height; BMI; parity	n (%): Unkown<br>Method: Complete-case analysis	Int: None (Apparent performance)<br>Ext : None	Cal: Not evaluated<br>Disc : C-Statistic / AUC graph<br>Ov : Not evaluated<br>Util: Not evaluated
Mendez-Figueroa, 2014	Treatment modality according to institutional protocol	Logistic regression	367 (143)	8	3	17.9	Based on univariable associations	Pre-specified model (not selection)	BMI at 26–30 weeks; GA at OGTT; fasting blood glucose at OGTT	n (%): Unkown<br>Method: Complete-case analysis	Int: None (Apparent performance)<br>Ext : None	Cal: Not evaluated<br>Disc : C-Statistic / AUC graph<br>Ov : Not evaluated<br>Util: Not evaluated

Author, Year	Outcome	Modelling method	Sample size (events)	No predictors		EPV	Selection of candidate predictors	Selection of final predictors	Predictors	Number (%) and handling of missing data	Type of validation	Performance measures
				Cand	Final							
Much, 2015	Treatment modality according to institutional protocol	Logistic Regression	856 (183)	Unkown	3	NI	NI	NI	Fasting glucose; BMI at OGTT; 1-h glucose	n (%): Unkown<br>Method: No Information	Int: None (Apparent performance)<br>Ext : None	Cal: Not evaluated<br>Disc : Not evaluated<br>Ov : Not evaluated<br>Util: Not evaluated
Muche, 2020	Impaired fasting glucose defined as FPG 100–125mg/dL; impaired glucose tolerance defined as 2-hour plasma glucose in 75g OGTT 140–199mg/ dL) or diabetes (FPG ≥126mg/dL, or 2-hour plasma glucose ≥200 mg/dL in OGTT or random plasma glucose ≥200mg/dL	Logistic regression	112 (24)	15	4	1.6	Based on univariable associations	Backward elimination	Maternal age; mid-upper arm circumference; FPG at GDM diagnosis; Antenatal depression	n (%): Unkown<br>Method: No information	Int: Bootstrap<br>Ext : None	Cal: Not evaluated<br>Disc : C-Statistic / AUC graph<br>Ov : Not evaluated<br>Util: Not evaluated
Mukherjee (a), 2023	Neonatal hypoglycemia (<2.6 mmol/L or 46.8 mg/dL in symptomatic and preterm babies; <2.0 mmol/L or 36 mg/dL in asymptomatic term babies)	Logistic Regression	627 (160)	3	3	53.3	All available predictors	Pre-specified model (not selection)	Age; BMI at first antenatal presentation; DRIPP score	n (%): 23 (3.7)<br>Method: Complete-case analysis	Int: None (Apparent performance)<br>Ext : None	Cal: Not evaluated<br>Disc : C-Statistic / AUC graph<br>Ov : Not evaluated<br>Util: Not evaluated
Mukherjee (b), 2023	Preterm delivery < 37 weeks	Logistic Regression	627 (52)	3	3	17.3	All available predictors	Pre-specified model (not selection)	Age; BMI at first antenatal presentation; DRIPP score	n (%): 23 (3.7)<br>Method: Complete-case analysis	Int: None (Apparent performance)<br>Ext : None	Cal: Not evaluated<br>Disc : C-Statistic / AUC graph<br>Ov : Not evaluated<br>Util: Not evaluated
Mukherjee (c), 2023	Hyperbilirubinemia based on variable diagnostic thresholds according to gestational and postnatal age	Logistic Regression	627 (26)	3	3	8.7	All available predictors	Pre-specified model (not selection)	Age; BMI at first antenatal presentation; DRIPP score	n (%): 23 (3.7)<br>Method: Complete-case analysis	Int: None (Apparent performance)<br>Ext : None	Cal: Not evaluated<br>Disc : C-Statistic / AUC graph<br>Ov : Not evaluated<br>Util: Not evaluated
Mukherjee (d), 2023	NICU admission	Logistic Regression	627 (71)	3	3	23.7	All available predictors	Pre-specified model (not selection)	Age; BMI at first antenatal presentation; DRIPP score	n (%): 23 (3.7)<br>Method: Complete-case analysis	Int: None (Apparent performance)<br>Ext : None	Cal: Not evaluated<br>Disc : C-Statistic / AUC graph<br>Ov : Not evaluated<br>Util: Not evaluated

Author, Year	Outcome	Modelling method	Sample size (events)	No predictors		EPV	Selection of candidate predictors	Selection of final predictors	Predictors	Number (%) and handling of missing data	Type of validation	Performance measures
				Cand	Final							
Mukherjee (e), 2023	Macrosomia defined as a birth weight ≥ 4000g	Logistic Regression	627 (30)	3	3	10.0	All available predictors	Pre-specified model (not selection)	Age; BMI at first antenatal presentation; DRIPP score	n (%): 23 (3.7) Method: Complete-case analysis	Int: None (Apparent performance) Ext : None	Cal: Not evaluated Disc : C-Statistic / AUC graph Ov : Not evaluated Util: Not evaluated
Mukherjee (f), 2023	Shoulder dystocia or birth injury	Logistic Regression	627 (4)	3	3	1.3	All available predictors	Pre-specified model (not selection)	Age; BMI at first antenatal presentation; DRIPP score	n (%): 23 (3.7) Method: Complete-case analysis	Int: None (Apparent performance) Ext : None	Cal: Not evaluated Disc : C-Statistic / AUC graph Ov : Not evaluated Util: Not evaluated
Mukherjee (g), 2023	Neonatal complication composite (composite of any of the six morbidity-related outcomes, within 30 days of birth)	Logistic Regression	627 (343)	3	3	94.7	All available predictors	Pre-specified model (not selection)	Age; BMI at first antenatal presentation; DRIPP score	n (%): 23 (3.7) Method: Complete-case analysis	Int: None (Apparent performance) Ext : None	Cal: Not evaluated Disc : C-Statistic / AUC graph Ov : Not evaluated Util: Not evaluated
Odinokova, 2019	Macrosomia defined as a birth weight ≥ 4000g	Machine learning techniques (OPLS-DA)	120 (40)	NI	13	NI	All available predictors	Other	Unclear but lipids	n (%): Unkown Method: No information	Int: None (Apparent performance) Ext : None	Cal: Not evaluated Disc : C-Statistic / AUC graph Ov : Not evaluated Util: Not evaluated
Park, 2015	Adverse outcomes(neonatal hypoglycemia, hyperbilirubinemia, and hyperinsulinemia; admission to the neonatal intensive care unit; large for gestational age; gestational insulin therapy; and gestational hypertension)	Logistic regression	802 (458)	9	2	38.2	Based on univariable associations	Pre-specified model (not selection)	BMI at entry and FBG	n (%): Unkown Method: No information	Int: None (Apparent performance) Ext : None	Cal: HL test Disc : C-Statistic / AUC graph Ov : Not evaluated Util: Not evaluated
Pei, 2023	Remission of impaired glucose toelrance defined as 3.9 mmol/L ≤ FPG < 6.1 mmol/L and 2 h PG < 7.8 mmol/L.	Logistic Regression	113 (55)	8	8	6.9	Unclear	Unclear	Age; income; education; GDM history; family diabetes history; pre-BMI; waist at recruitment, PANDER level	n (%): Unkown Method: No information	Int: None (Apparent performance) Ext : None	Cal: Not evaluated Disc : C-Statistic / AUC graph Ov : Not evaluated Util: Not evaluated
				27	16	7.9						

Author, Year	Outcome	Modelling method	Sample size (events)	No predictors		EPV	Selection of candidate predictors	Selection of final predictors	Predictors	Number (%) and handling of missing data	Type of validation	Performance measures
				Cand	Final							
Periyathambi, 2022	Non-attendance at postpartum glucose test (OGTT 6-12 weeks post delivery, HbA1C 3 months post delivery)	Logistic regression	607 (213)				All available predictors	LASSO selection	Maternal age; antenatal FPG, 2hrs, Hb1Ac; gestational age at diagnosis; BMI; smoker; unmarried; diastolic BP; Ethnicity; Gestational age at birth, Instrument assisted delivery, SGA infants, male babies, Breastfeeding before discharge, Parity	n (%): 133 (21.9)<br>Method: Multiple imputation	Int: Cross-validation<br>Ext : None	Cal: Not evaluated<br>Disc : C-Statistic / AUC graph<br>Ov : Not evaluated<br>Util: DCA
Phaloprakarn, 2009	Preeclampsia diagnosed using the criteria of the International Society for the Study of Hypertension in Pregnancy	Logistic regression	813 (78)	11	3	7.1	Based on univariable associations	Pre-specified model (not selection)	First-trimester BMI; GA at GDM diagnosis; Poor glycemic control	n (%): Unkown<br>Method: No information	Int: None (Apparent performance)<br>Ext : None	Cal: HL test<br>Disc : C-Statistic / AUC graph<br>Ov : Not evaluated<br>Util: Not evaluated
Phaloprakarn (a), 2020	Primary caesarean delivery	Logistic regression	385 (130)	5	3	26.0	Based on univariable associations	Pre-specified model (not selection)	Parity; Gestational weight gain; Insulin Use	n (%): 3 (0.8)<br>Method: Multiple imputation	Int: None (Apparent performance)<br>Ext : Temporal	Cal: Calibration plot / HL test<br>Disc : C-Statistic / AUC graph<br>Ov : Not evaluated<br>Util: Not evaluated
Phaloprakarn (b), 2020	Primary caesarean delivery	Logistic Regression	448 (178)	NA	3	NA	NA	NA	Parity; Gestational weight gain; Insulin Use	n (%): Unkown<br>Method: No information	Int: NA<br>Ext : NA	Cal: Calibration plot / HL test<br>Disc : C-Statistic / AUC graph<br>Ov : Not evaluated<br>Util: Not evaluated
Pintaudi, 2018	Neonatal adverse outcome (fetal growth, LGA, SGA mortality, malformations, shoulder distocia, neonatal intensive care need, hypoglycaemia, hypocalcemia, hyperbilirubinemia, and respiratory distress	Machine learning techniques (RECPAM)	2,736 (815)	7	3	116.4	Based on prior knowledge	Other	Pre-pregnancy BMI; Pre-pregnancy BMI; family history of diabetes	n (%): Unkown<br>Method: No information	Int: None (Apparent performance)<br>Ext : None	Cal: Not evaluated<br>Disc : Not evaluated<br>Ov : Not evaluated<br>Util: Not evaluated
Ramos, 2023				24	8	41.1						

Author, Year	Outcome	Modelling method	Sample size (events)	No predictors		EPV	Selection of candidate predictors	Selection of final predictors	Predictors	Number (%) and handling of missing data	Type of validation	Performance measures
				Cand	Final							
	Primary caesarean delivery	Logistic Regression	3,570 (987)				Based on prior knowledge	Stepwise backward elimination	Insulin required in pregnancy; Nulliparous; Preeclampsia; Polyhydramnios; Large for gestational age; Age at delivery; Early pregnancy body mass index; Hemoglobin A1C	n (%): Unkown<br>Method: Complete-case analysis	Int: Bootstrap<br>Ext : None	Cal: Calibration plot / Slope / CITL / HL test<br>Disc : C-Statistic / AUC graph<br>Ov : R-squared<br>Util: Not evaluated
Schwartz, 2016	GDM recurrence (GDM in a pregnancy following the index pregnancy complicated by GDM)	Logistic regression	788 (432)	25	9	14.2	Based on univariable associations	Pre-specified model (not selection)	Maternal age; multiparity; GDM diagnosis week; fasting OGTT; OGTT after 1 h; OGTT after 2 hrs; insulin use; inter-pregnancy interval; BMI gain	n (%): Unkown<br>Method: Complete-case analysis	Int: None (Apparent performance)<br>Ext : None	Cal: HL test<br>Disc : Not evaluated<br>Ov : Not evaluated<br>Util: Not evaluated
Souza, 2018	When glycemic targets were not achieved with diet and exercise (fasting >95mg/dL and 1 h post-prandial >140 mg/dL), insulin therapy was initiated along with continuing the dietary treatment.	Logistic regression	408 (135)	13	5	10.4	Based on prior knowledge	Stepwise selection	Previous GDM; family history of DM; BMI; age; FBG	n (%): Unkown<br>Method: No information	Int: Cross-validation<br>Ext : None	Cal: Not evaluated<br>Disc : C-Statistic<br>Ov : Not evaluated<br>Util: Not evaluated
Sun, 2020	Macrosomia defined as a birth weight ≥ 4000g	Logistic Regression	64 (12)	8	5	1.5	Based on univariable associations	Other	Prepregnancy BMI; Weight gain; C0; C16; C3 (cartinine metabolites)	n (%): Unkown<br>Method: No Information	Int: No Information<br>Ext : None	Cal: Not evaluated<br>Disc : C-Statistic / AUC graph<br>Ov : Not evaluated<br>Util: Not evaluated
Sun, 2023	Macrosomia defined as a birth weight ≥ 4000g	Logistic Regression	991 (73)	20	5	3.7	Based on univariable associations	Stepwise selection	Gravidity; pre-pregnancy body mass index; family history of hypertension; abdominal circumference; and biparietal diameter	n (%): Unkown<br>Method: No information	Int: Random split data<br>Ext : None	Cal: Calibration plot / Slope / CITL / HL test<br>Disc : C-Statistic / AUC graph<br>Ov : Not evaluated<br>Util: DCA
Tomlinson, 2018	Fetal overgrowth defined as a birthweight that is ≥ 90th gestation-related optimal weight (GROW) centile	Logistic regression	275 (51)	24	5	2.1	Based on univariable associations	Forward selection	Age; history of macrosomia; excessive gestational weight gain; enlarged fetal abdominal circumference; and high fasting glucose	n (%): Unkown<br>Method: No information	Int: Bootstrap<br>Ext : None	Cal: Not evaluated<br>Disc : C-Statistic / AUC graph<br>Ov : Not evaluated<br>Util: Not evaluated
Tomlinson, 2021	As above	Logistic regression	477 (100)	NA	5	NA	NA	NA	As above	n (%): Unkown<br>Method: Other	Int: NA<br>Ext : NA	Cal: Not evaluated<br>Disc : C-Statistic<br>Ov : Not evaluated<br>Util: Not evaluated

Author, Year	Outcome	Modelling method	Sample size (events)	No predictors		EPV	Selection of candidate predictors	Selection of final predictors	Predictors	Number (%) and handling of missing data	Type of validation	Performance measures
				Cand	Final							
Ukah (a), 2022	Hospitalization for type 2 diabetic complications within 10 years after delivery of the first pregnancy affected by GDM. Type 2 diabetic complications was defined as a diagnosis of type 2 diabetes with the development of one or more of the following complications: diabetic coma, acidosis, kidney, ophthalmic, neurological, circulatory, or other complications resulting from diabetes.	Cox regression	90,143 (283)	13	7	21.8	Based on prior knowledge	LASSO selection	Maternal age; Socioeconomically deprived; Substance use; Gestational age at delivery; Severe maternal morbidity; Previous complications; Hypertensive disorders of pregnancy	n (%): Unkown<br>Method: No information	Int: Bootstrap<br>Ext : None	Cal: Calibration plot / Slope / CITL<br>Disc : C-Statistic / AUC graph<br>Ov : Not evaluated<br>Util: Not evaluated
Ukah (b), 2022	Hospitalization for type 2 diabetic complications within 29 years after delivery of the first pregnancy affected by GDM.	Cox regression	90,143 (1025)	13	7	78.8	Based on prior knowledge	LASSO selection	Maternal age; Socioeconomically deprived; Substance use; Gestational age at delivery; Severe maternal morbidity; Previous complications; Hypertensive disorders of pregnancy	n (%): Unkown<br>Method: No information	Int: Bootstrap<br>Ext : None	Cal: Not evaluated<br>Disc : C-Statistic<br>Ov : Not evaluated<br>Util: Not evaluated
Velardo, 2021	Need for pharmacological treatment (metformin and/or insulin) according to institutional protocol	Logistic regression	1,543 (454)	28	NI	16.2	Based on prior knowledge	LASSO selection	NI	n (%): Unkown<br>Method: Single imputation	Int: Random split data<br>Ext : None	Cal: Not evaluated<br>Disc : C-Statistic / AUC graph<br>Ov : Not evaluated<br>Util: Not evaluated
Yerlikaya (a), 2018	Need for pharmacological treatment according to institutional protocol	Logistic regression	203 (95)	3	3	31.7	All available predictors	Pre-specified model (not selection)	OGTT glucose measurements at fasting; 60min; and 120min	n (%): Unkown<br>Method: Multiple imputation	Int: None (Apparent performance)<br>Ext : None	Cal: Not evaluated<br>Disc : C-Statistic / AUC graph<br>Ov : Not evaluated<br>Util: Not evaluated
Yerlikaya (b), 2018	Need for pharmacological treatment according to institutional protocol	Logistic regression	203 (95)	6	6	15.8	All available predictors	Pre-specified model (not selection)	Age; pregestational BMI; parity; history of GDM; family history with type 2 diabetes; and time of diagnosis	n (%): Unkown<br>Method: Multiple imputation	Int: None (Apparent performance)<br>Ext : None	Cal: Not evaluated<br>Disc : C-Statistic / AUC graph<br>Ov : Not evaluated<br>Util: Not evaluated

Author, Year	Outcome	Modelling method	Sample size (events)	No predictors		EPV	Selection of candidate predictors	Selection of final predictors	Predictors	Number (%) and handling of missing data	Type of validation	Performance measures
				Cand	Final							
Yerlikaya (c), 2018	Need for pharmacological treatment according to institutional protocol	Logistic regression	203 (95)	9	9	10.6	All available predictors	Pre-specified model (not selection)	OGTT glucose measurements at fasting; 60min; and 120min; age; pregestational BMI; parity; history of GDM; family history of diabetes; and time of diagnosis	n (%): Unkown<br>Method: Multiple imputation	Int: None (Apparent performance)<br>Ext : None	Cal: Not evaluated<br>Disc : C-Statistic / AUC graph<br>Ov : Not evaluated<br>Util: Not evaluated
Yuan, 2022	Macrosomia defined as a birth weight ≥ 4000g	Logistic regression	40 (20)	NI	5	NI	Based on univariable associations	Pre-specified model (not selection)	Maternal age; fasting plasma glucose; 2-h plasma glucose; GDF3 expression; AC006064.4 expression in peripheral blood exosomes	n (%): Unkown<br>Method: No information	Int: None (Apparent performance)<br>Ext : None	Cal: HL test<br>Disc : C-Statistic / AUC graph<br>Ov : Not evaluated<br>Util: Not evaluated
Yuan, 2023	Macrosomia defined as a birth weight ≥ 4000g	Logistic Regression	88 (44)	12	9	3.7	Based on univariable associations	LASSO selection	Pre-pregnancy BMI; weight gain; OGTT 2h glucose; parity; HDL; LDL; plasma CLUL1 expression; plasma VCAN expression; plasma RNASE3 expression	n (%): Unkown<br>Method: Other	Int: Cross-validation<br>Ext : None	Cal: Calibration plot / HL test<br>Disc : C-Statistic / AUC graph<br>Ov : Not evaluated<br>Util: Clinical impact curve
Zaccara, 2023	Treatment modality according to institutional protocol	Logistic Regression	869 (145)	6	6	24.2	Based on univariable associations	Other	Previous GDM; BMI; age; FPG OGTT; 1hPG; 2hPG	n (%): Unkown<br>Method: No information	Int: None (Apparent performance)<br>Ext : None	Cal: Not evaluated<br>Disc : C-Statistic / AUC graph<br>Ov : Not evaluated<br>Util: Not evaluated
Zhang, 2021	Type 2 Diabetes diagnosed based on American Diabetes Association criteria	Machine learning techniques (Random Forest)	216 (98)	69	10	1.4	Based on univariable associations	Decision Trees	AC16; Orn; PC aa C30:0; Putrescine; Val; PC ae C32:2; SM (OH) C22:2; Spermidine; Phe; lyso PC a C28:0	n (%): Unkown<br>Method: Single imputation	Int: Cross-validation<br>Ext : None	Cal: Not evaluated<br>Disc : C-Statistic / AUC graph<br>Ov : Not evaluated<br>Util: Not evaluated
Zhang, 2023	Macrosomia defined as a birth weight ≥ 4000g	Logistic Regression	322 (21)	11	4	1.9	Based on univariable associations	Other	Pregnancy gain weight; Amniotic fluid index; OGTT Fasting glucose; Fetal weight estimated by ultrasound	n (%): Unkown<br>Method: Complete-case analysis	Int: None (Apparent performance)<br>Ext : None	Cal: Not evaluated<br>Disc : C-Statistic / AUC graph<br>Ov : Not evaluated<br>Util: Not evaluated
Zou, 2021	Macrosomia defined as a birth weight ≥ 4000g	Logistic Regression	783 (83)	21	6	4.0	Based on univariable associations	Forward selection	Body mass index; weight gain during pregnancy; fasting plasma glucose; triglycerides; biparietal diameter; amniotic fluid index	n (%): Unkown<br>Method: No information	Int: Bootstrap<br>Ext : None	Cal: Calibration plot<br>Disc : C-Statistic / AUC graph<br>Ov : Not evaluated<br>Util: Not evaluated

Abbreviations: No information (NI); Area under the curve (AUC); Calibration in the large (CITL); Hosmer-Lemeshow (HL); Classification and Regression Trees (CART); Artificial Immune Recognition System (AIRS); Orthogonal Partial Least Squares Discriminant Analysis (OPLS-DA); REcursive Partition and Amalgamation (RECPAM)

Completed information on data extraction using CHARMS and critical appraisal PROBAST for each study is available upon request

Supplementary Table 4. Critical appraisal and applicability assessment of included models

Author, Year		Critical appraisal (PROBAST)				
		P	Pr	O	A	Overall
Allalou (a), 2016	RoB	-	+	+	-	-
	App	-	-	+		-
Allalou (b), 2016	RoB	-	+	+	-	-
	App	-	+	+		-
Allalou (c), 2016	RoB	-	+	+	-	-
	App	-	-	+		-
Badr (a), 2022	RoB	-	+	+	-	-
	App	-	+	+		-
Badr (b), 2022	RoB	-	+	+	-	-
	App	-	+	+		-
Badr (c), 2022	RoB	-	+	+	-	-
	App	-	+	+		-
Badr (d), 2022	RoB	-	+	+	-	-
	App	-	+	+		-
Barden, 2013	RoB	+	+	-	-	-
	App	+	+	+		+
Barnes (a), 2016	RoB	-	+	-	-	-
	App	+	+	+		+
Barnes (b), 2016	RoB	?	+	-	-	-
	App	?	+	+		?

Author, Year		Critical appraisal (PROBAST)				
		P	Pr	O	A	Overall
Bartakova, 2021	RoB	+	+	+	-	-
	App	-	+	+		-
Bengtson, 2022	RoB	+	+	+	-	-
	App	+	+	+		+
Cooray (a), 2022	RoB	+	+	+	+	+
	App	+	+	+		+
Cooray (b), 2022	RoB	+	+	+	+	+
	App	+	+	+		+
Cormier (a), 2014	RoB	-	-	+	-	-
	App	-	-	+		-
Cormier (b), 2014	RoB	-	-	+	-	-
	App	-	-	+		-
Du, 2021	RoB	-	+	-	-	-
	App	-	+	+		-
Eleftheriades (a), 2021	RoB	+	+	-	-	-
	App	+	+	+		+
Eleftheriades (b), 2021	RoB	?	?	-	-	-
	App	?	?	?		?
Elnour, 2006	RoB	?	-	?	-	-
	App	?	-	?		-

Author, Year		Critical appraisal (PROBAST)				
		P	Pr	O	A	Overall
Ford, 2022	RoB	+	+	-	+	-
	App	+	+	-		-
Hahn, 2023	RoB	+	-	+	-	-
	App	+	+	+		+
Harper, 2016	RoB	-	+	+	-	-
	App	-	+	+		-
Huang (a), 2023	RoB	+	+	+	-	-
	App	+	+	+		+
Huang (b), 2023	RoB	+	+	+	-	-
	App	+	+	+		+
Ignell (a), 2016	RoB	+	-	-	-	-
	App	+	-	-		-
Ignell (b), 2016	RoB	+	-	-	-	-
	App	+	-	-		-
Ingram (a), 2017	RoB	+	-	+	-	-
	App	+	+	+		+
Ingram (b), 2017	RoB	+	-	?	-	-
	App	+	+	?		?
Ingram (c), 2017	RoB	+	-	?	-	-
	App	+	+	?		?

Author, Year		Critical appraisal (PROBAST)				
		P	Pr	O	A	Overall
Joglekar (a), 2021	RoB	+	-	+	-	-
	App	+	+	+		+
Joglekar (b), 2021	RoB	+	-	+	-	-
	App	+	-	+		-
Jotic, 2023	RoB	-	-	+	-	-
	App	-	+	+		-
Kang (a), 2019	RoB	-	-	+	-	-
	App	+	+	+		+
Kang (b), 2019	RoB	-	-	+	-	-
	App	+	+	+		+
Khan, 2019	RoB	-	+	+	-	-
	App	-	-	+		-
Kim, 2022	RoB	+	-	+	-	-
	App	+	+	+		+
Koefoed, 2023	RoB	+	+	+	-	-
	App	+	+	+		+
Kohler, 2016	RoB	+	+	-	-	-
	App	+	+	+		+
Kondo (a), 2018	RoB	+	-	+	-	-
	App	+	+	+		+

Author, Year		Critical appraisal (PROBAST)				
		P	Pr	O	A	Overall
Kondo (b), 2018	RoB	+	-	+	-	-
	App	+	+	+		+
Kondo (c), 2018	RoB	+	-	+	-	-
	App	+	+	+		+
Kwak (a), 2013	RoB	+	+	+	-	-
	App	-	+	+		-
Kwak (b), 2013	RoB	+	+	+	-	-
	App	-	-	+		-
Kwak (c), 2013	RoB	+	+	+	-	-
	App	-	+	+		-
Kwak (d), 2013	RoB	+	+	+	-	-
	App	-	-	+		-
Lai, 2020	RoB	-	+	+	-	-
	App	-	-	+		-
Lappas (a), 2015	RoB	+	+	+	-	-
	App	+	+	+		+
Lappas (b), 2015	RoB	+	+	+	-	-
	App	+	-	+		-
Lappas (c), 2015	RoB	+	+	+	-	-
	App	+	-	+		-

Author, Year		Critical appraisal (PROBAST)				
		**P**	**Pr**	**O**	**A**	**Overall**
Lappas (a), 2016	**RoB**	+	+	+	-	-
	**App**	+	+	+		+
Lappas (b), 2016	**RoB**	+	+	+	-	-
	**App**	+	-	+		-
Lappas (c), 2016	**RoB**	+	+	+	-	-
	**App**	+	-	+		-
Lappas (a), 2018	**RoB**	+	+	+	-	-
	**App**	+	+	+		+
Lappas (b), 2018	**RoB**	+	+	+	-	-
	**App**	+	-	+		-
Lappas (c), 2018	**RoB**	+	+	+	-	-
	**App**	+	-	+		-
Liao (a), 2022	**RoB**	+	+	+	+	+
	**App**	+	+	+		+
Liao (b), 2022	**RoB**	+	+	+	+	+
	**App**	+	+	+		+
Liao (c), 2022	**RoB**	+	+	+	+	+
	**App**	+	+	+		+
Liao (d), 2022	**RoB**	+	+	+	+	+
	**App**	+	+	+		+

Author, Year		Critical appraisal (PROBAST)				
		P	Pr	O	A	Overall
Liao (e), 2022	RoB	+	+	+	+	+
	App	+	+	+		+
Liao (f), 2022	RoB	+	+	+	+	+
	App	+	+	+		+
Liao (g), 2022	RoB	+	+	+	+	+
	App	+	+	+		+
Liao (h), 2022	RoB	+	+	+	+	+
	App	+	+	+		+
Lin, 2011	RoB	?	?	+	-	-
	App	?	?	+		?
Li (a), 2018	RoB	-	-	+	-	-
	App	+	+	+		+
Li (b), 2018	RoB	-	-	+	-	-
	App	+	+	+		+
Lu (a), 2022	RoB	+	+	+	-	-
	App	+	+	+		+
Lu (b), 2022	RoB	+	+	+	-	-
	App	+	-	+		-
Man, 2021	RoB	+	+	+	-	-
	App	-	+	+		-

Author, Year		Critical appraisal (PROBAST)				
		P	Pr	O	A	Overall
Marozas (a), 2018	RoB	?	?	?	-	-
	App	?	-	?		-
Marozas (b), 2018	RoB	?	?	?	-	-
	App	?	-	?		-
McIntyre (a), 2018	RoB	+	+	+	-	-
	App	-	+	+		-
McIntyre (b), 2018	RoB	+	+	+	-	-
	App	-	+	+		-
McIntyre (c), 2018	RoB	+	+	+	-	-
	App	-	+	+		-
McIntyre (d), 2018	RoB	+	+	+	-	-
	App	-	+	+		-
McIntyre (e), 2018	RoB	+	+	+	-	-
	App	-	+	+		-
McIntyre (f), 2018	RoB	+	+	+	-	-
	App	-	+	+		-
Mendez-Figueroa, 2014	RoB	+	+	+	-	-
	App	-	+	+		-
Much, 2015	RoB	?	+	+	-	-
	App	?	+	+		?

Author, Year		Critical appraisal (PROBAST)				
		P	Pr	O	A	Overall
Muche, 2020	RoB	+	+	+	-	-
	App	-	+	+		-
Mukherjee (a), 2023	RoB	?	-	+	-	-
	App	?	+	+		?
Mukherjee (b), 2023	RoB	?	-	+	-	-
	App	?	+	+		?
Mukherjee (c), 2023	RoB	?	-	+	-	-
	App	?	+	+		?
Mukherjee (d), 2023	RoB	?	-	+	-	-
	App	?	+	+		?
Mukherjee (e), 2023	RoB	?	-	+	-	-
	App	?	+	+		?
Mukherjee (f), 2023	RoB	?	-	+	-	-
	App	?	+	+		?
Mukherjee (g), 2023	RoB	?	-	+	-	-
	App	?	+	+		?
Odinokova, 2019	RoB	+	+	+	-	-
	App	-	-	+		-
Park, 2015	RoB	+	-	+	-	-
	App	-	+	+		-

Author, Year		Critical appraisal (PROBAST)				
		P	Pr	O	A	Overall
Pei, 2023	RoB	-	+	+	-	-
	App	?	+	+		?
Periyathambi, 2022	RoB	+	+	+	-	-
	App	+	+	+		+
Phaloprakarn, 2009	RoB	+	+	+	-	-
	App	+	+	+		+
Phaloprakarn (a), 2020	RoB	+	+	+	+	+
	App	+	+	+		+
Phaloprakarn (b), 2020	RoB	+	+	+	+	+
	App	+	+	+		+
Pintaudi, 2018	RoB	+	+	+	-	-
	App	+	+	+		+
Ramos, 2023	RoB	+	+	+	+	+
	App	+	+	+		+
Schwartz, 2016	RoB	+	-	+	-	-
	App	+	+	+		+
Souza, 2018	RoB	+	+	-	-	-
	App	+	+	+		+
Sun, 2020	RoB	?	+	+	-	-
	App	?	-	+		-

Author, Year		Critical appraisal (PROBAST)				
		P	Pr	O	A	Overall
Sun, 2023	RoB	+	+	+	-	-
	App	+	+	+		+
Tomlinson, 2018	RoB	+	+	+	-	-
	App	+	+	+		+
Tomlinson, 2021	RoB	+	+	+	-	-
	App	+	+	+		+
Ukah (a), 2022	RoB	+	+	+	+	+
	App	+	+	+		+
Ukah (b), 2022	RoB	+	+	+	+	+
	App	+	+	+		+
Velardo, 2021	RoB	+	+	-	-	-
	App	+	+	+		+
Yerlikaya (a), 2018	RoB	+	+	-	-	-
	App	+	+	+		+
Yerlikaya (b), 2018	RoB	+	+	-	-	-
	App	+	+	+		+
Yerlikaya (c), 2018	RoB	+	+	-	-	-
	App	+	+	+		+
Yuan, 2022	RoB	?	-	+	-	-
	App	+	-	+		-

Author, Year		Critical appraisal (PROBAST)				
		**P**	**Pr**	**O**	**A**	**Overall**
Yuan, 2023	**RoB**	-	+	+	-	-
	**App**	-	-	+		-
Zaccara, 2023	**RoB**	+	+	-	-	-
	**App**	+	+	+		+
Zhang, 2021	**RoB**	+	+	+	-	-
	**App**	-	-	+		-
Zhang, 2023	**RoB**	-	+	+	-	-
	**App**	-	+	+		-
Zou, 2021	**RoB**	+	+	+	-	-
	**App**	+	+	+		+

Abbreviations: Prediction model Risk Of Bias ASsessment Tool (PROBAST); Population (P); Predictors (Pr); Outcome (O); Analysis (A); Risk of Bias (RoB); Applicability (App)

Completed information on data extraction using CHARMS and critical appraisal PROBAST for each study is available upon request

Supplementary Box 1. Prediction model for adverse pregnancy outcomes

To facilitate the identification of women at high risk of adverse pregnancy outcomes, Cooray et al. developed an individualised risk prediction model for a composite of GDM outcomes. The model was developed in a diverse population using routinely available predictors. Candidate predictors were selected based on prior studies rather than univariable selection, and categorisation of continuous variables was avoided. Missing data was adequately reported and handled with multiple imputation. The model was internally validated using bootstrap and externally validated using temporal validation. Model discrimination was evaluated using the C-statistic and calibration was evaluated with the calibration plot, calibration slope and calibration-in-the-large. Clinical utility was investigated using DCA. The full model equation was reported to enable independent validation, and an online calculator was provided to encourage clinical uptake. Cooray et al. followed a prospectively published protocol and the transparent reporting of a multivariable prediction model for individual prognosis or diagnosis (TRIPOD) guidelines.

Supplementary Box 2. Prediction model for type 2 diabetic complications

Ukah et al. developed a prediction model for type 2 diabetic complications in patients with GDM. The authors used Cox-proportional hazard models to account for loss to follow-up. Candidate predictors, routinely available in clinical practice, were selected based on prior knowledge and continuous variables were modelled using restricted cubic splines. The final variables were selected using LASSO. The model was assessed for internal validity with bootstrap and optimism was reported. Ukah et al. evaluated calibration using the calibration plot, slope and calibration-in-the large; and model discrimination using the C-statistic and AUC curve. Finally, the full model equation was presented, and the authors followed the TRIPOD guidelines.
